# Evaluation of the efficacy and safety of first-line therapies for peripheral T-cell lymphoma: a systematic review and network meta-analysis

**DOI:** 10.3389/fonc.2026.1912842

**Published:** 2026-08-24

**Authors:** Jiaxing Zhao, Bailin Wu, Youchao Jia

**Affiliations:** 1 Department of Medical Oncology, Affiliated Hospital of Hebei University, Baoding, Hebei, China; 2 West Campus of Hebei North University, Zhangjiakou, Hebei, China; 3 Department of Medical Oncology, Affiliated Hospital of Hebei University, Hebei Key Laboratory of Cancer Radiotherapy and Chemotherapy, Baoding, Hebei, China

**Keywords:** first-line treatment, network meta-analysis, os, peripheral T-cell lymphoma, PFS, subgroup analysis

## Abstract

**Objective:**

To compare the efficacy and safety of first-line therapies for peripheral T-cell lymphoma (PTCL) via network meta-analysis.

**Methods:**

We systematically searched PubMed, Embase, Cochrane Library, and Web of Science for randomized controlled trials (RCTs) evaluating first-line treatments in untreated PTCL patients (up to March 1, 2025). Two investigators independently screened studies, extracted data, and assessed bias risk. Network meta-analysis was performed using R (v4.3.0).

**Results:**

Seven RCTs (1,334 patients) were included, evaluating CHOP, VIP-rABVD, GEM-P, A+CHP, GDPT, Ro-CHOP, and ICED. No significant differences were found in overall or progression-free survival. However, A+CHP (OR = 1.90, 95%CI:1.20–3.10) and GDPT (OR = 2.00, 95%CI:1.00–3.80) showed higher objective response rates versus CHOP. A+CHP (OR = 1.76, 95%CI:1.09–2.88) and Ro-CHOP (OR = 1.40, 95%CI:1.00–1.80) achieved better disease control. VIP-rABVD had the highest thrombocytopenia risk (OR = 50.0, 95%CI:14.29–100), while ICED showed increased anemia risk (OR = 9.20, 95%CI:2.80–44.0). Subgroup analyses revealed no significant PFS differences in AITL/PTCL-NOS.

**Conclusion:**

A+CHP shows superior efficacy with acceptable toxicity, while GDPT improves responses but increases myelosuppression risk. Ro-CHOP enhances disease control but with limited remission depth and higher toxicity. Subtype analyses suggest potential PFS benefits for Ro-CHOP in AITL. For PTCL of unspecified type (PTCL-NOS), there is little difference in efficacy among the various treatment regimens.A+CHP and GDPT offer optimal benefit-risk profiles.

## Introduction

Peripheral T-cell lymphomas (PTCLs) are a heterogeneous group of aggressive non-Hodgkin lymphomas (NHLs) derived from post-thymic mature T lymphocytes. Major subtypes include PTCL-not otherwise specified (PTCL-NOS), angioimmunoblastic T-cell lymphoma (AITL), and anaplastic large-cell lymphoma (ALCL) categorized by anaplastic lymphoma kinase (ALK)-positive or negative (1). PTCLs predominantly occur in older adults over 60 years, with a slight predominance in males (male-to-female ratio of nearly 1.3:1). Accounting for 15%-20% of aggressive NHL cases, PTCLs are less prevalent than B-cell lymphomas (2, 3) but tend to exhibit greater clinical aggressiveness, rapid disease progression, suboptimal responses to conventional chemotherapy, and high relapse rates. The 5-year overall survival (OS) approaches 30%, except for ALK-positive ALCL, which shows a significantly better prognosis (5-year OS of 70%–90%) (4). Traditional first-line therapy for PTCLs primarily involves anthracycline-based regimens like CHOP or CHOEP (CHOP plus etoposide) (5), extrapolated from early trials targeting PTCLs alongside aggressive B-cell NHLs (6). While effective for diffuse large B-cell lymphoma (DLBCL), these regimens yield limited long-term efficacy in most PTCL patients, especially those with non-ALCL subtypes. Therefore, the role of anthracyclines in PTCL management remains debated (7).

Recent advances, including intensified chemotherapy, targeted agents, and immunotherapies, have improved outcomes in select PTCL subgroups. Autologous stem cell transplantation (ASCT) offers superior efficacy over chemotherapy alone and is recommended for younger, treatment-naïve patients that suffer from high-risk features and chemosensitive disease. Nonetheless, ASCT’s benefits vary by subtype (being more effective in AITL and ALK-ALCL, but limited in PTCL-NOS) (8, 9). The ECHELON-2 trial revealed that the A+CHP regimen (brentuximab vedotin [BV] combined with cyclophosphamide, doxorubicin, and prednisone) led to a great improvement of progression-free survival (PFS) and OS in CD30-positive PTCL subtypes compared to CHOP. Nonetheless, this benefit appears attenuated in patients with low CD30 expression (10). Histone deacetylase (HDAC) inhibitors (e.g., romidepsin, belinostat) may enhance response rates but are associated with elevated risks of cardiac toxicity and infection. PI3K inhibitors (e.g., duvelisib) have an objective response rate (ORR) of about 50%, though their immune-related toxicity requires further investigation on efficacy and safety (3); JAK/STAT inhibitors show potential in patients with STAT3 mutations, but remain in early-phase clinical development (11). Emerging combination strategies integrating chemotherapy, immunotherapy, and novel targeted agents hold promise for synergistic efficacy. However, optimal combination regimens require further validation in phase III clinical trials (12).

Due to the rarity and heterogeneity of PTCLs, conventional therapies like CHOP or CHOEP face significant limitations. Emerging therapeutic strategies remain largely exploratory, and large-scale, randomized, prospective clinical trials are scarce. Consequently, no consensus exists on the optimal first-line therapy for PTCLs patients (13). Although a growing body of research has focused on first-line treatment of PTCLs, most existing studies are single-center retrospective analyses characterized by small sample sizes and high heterogeneity. Moreover, these studies often lack adequate subgroup analyses (14), and considerable variability exists among study findings. Direct head-to-head comparisons among frontline therapeutic regimens are scarce. Therefore, a comprehensive comparison of different frontline regimens is urgently needed. This study seeks to perform a systematic review and network meta-analysis (NMA) to evaluate and contrast the efficacy and safety of first-line therapies for PTCLs. By synthesizing data from randomized controlled trials (RCTs) and using the CHOP regimen as a reference, this study implemented exploratory subgroup analyses on major PTCL subtypes. The goal is to elucidate survival benefits and safety profiles across regimens, and support the development of optimized clinical guidelines for PTCLs, thereby enabling healthcare providers to make informed, evidence-based treatment choices aligned with individual patient characteristics, improving therapeutic outcomes, reducing adverse events (AEs), and enhancing patients’ quality of life.

## Methods and materials

This systematic review and meta-analysis followed PRISMA 2020 reporting guideline. A complete *a priori* review protocol was drafted before literature screening and archived publicly on Zenodo (DOI: 10.5281/zenodo.20740052). Protocol registration on PROSPERO was not completed prior to data extraction, which is acknowledged as a limitation of this work.

### Literature search

A comprehensive search was conducted in the Cochrane Library, PubMed, Embase, and Web of Science databases to identify the RCTs investigating the efficacy and safety of first-line treatments in newly diagnosed PTCL patients. The final search date was March 1, 2025. A combination of subject terms and free-text keywords was used, including Lymphoma, T-Cell, Peripheral, Peripheral T-Cell Lymphoma, newly diagnosed, untreated, first-line, front-line, and initial. The full search strategy is detailed in **Supplementary Material 1**.

### Study selection

A stepwise de-duplication and screening process was implemented. Duplicate entries were first removed using EndNote software, followed by manual review to eliminate residual duplicates. Non-eligible studies that did not involve newly diagnosed PTCL patients were excluded systematically. The remaining potentially eligible studies were then screened via a three-step process: title screening, abstract review, and full-text assessment. Two investigators initiated the entire screening process independently. Discrepancies were settled by consensus meetings to ensure objectivity and accuracy.

### Inclusion and exclusion criteria

Inclusion criteria: (1) Adult patients had confirmed diagnosis of PTCL according to recognized diagnostic standards; (2) Intervention group received first-line treatments (e.g., VIP-rABVD, GEM-P, A+CHP, GDPT, Ro-CHOP, ICED); (3) Control group received conventional CHOP regimen; (4) Primary outcomes included OS, PFS, and AE; (5) Secondary outcomes included ORR, disease control rate (DCR), PFS in AITL and PTCL-NOS subgroups; (5) Study design restricted to RCTs.

Exclusion criteria: (1) Animal studies, cell-based experiments, study protocols, case reports, reviews, letters, editorials, and conference abstracts; (2) Studies with incomplete data or serious methodological errors; (3) Duplicate publications; (4) Review articles; (5) Studies without accessible full text; (6) Studies involving patients with comorbid organic diseases or mixed cohorts.

### Data extraction

Literature screening was independently conducted by two investigators based on the pre-defined inclusion and exclusion criteria. Any discrepancies that arose during the data extraction process were addressed through discussion or, if necessary, resolved by consulting a third party. The extracted data were organized using Microsoft Excel 2021 and encompassed the following key details: first author, publication year, country, sample size, gender distribution, age, intervention regimens, follow-up duration, and outcome indicators. The data extraction and analysis process comprised: (1) Direct extraction of raw data from the articles; (2) Extraction of survival data from Kaplan–Meier curves via Engauge Digitizer software (a method validated by authoritative methodology (15)); (3) Transformation of extracted data into logarithmic hazard ratios (lnHR) and standard errors (following guidance from established methodological references (16–18).

### Quality assessment

The Cochrane Risk of Bias 2.0 (ROB 2.0) tool (19) was employed to evaluate the risk of bias for each included study (19) across five key areas: deviation in the randomization process, deviation in intended interventions, missing outcome data, outcome measurement, and selective reporting. Studies were categorized as “low risk of bias”, “unclear risk of bias”, or “high risk of bias”. Two investigators cross-verified the assessments, with any discrepancies addressed through discussion or, if needed, resolved by consulting a third party.

### Data analysis

All graphs were generated using R version 4.3.0 (R Foundation for Statistical Computing) and Stata 15.0 software. A Bayesian analytical framework with random-effects modeling was implemented to synthesize evidence from RCTs, with a view to accounting for parameter heterogeneity across studies. The Markov Chain Monte Carlo (MCMC) simulation method was used, with four independent chains running in parallel for 50,000 iterations each (the first 20,000 iterations were used for model calibration). This approach allowed the estimation of treatment effects and probability distributions (20–22). Convergence was evaluated through trace plots and Brooks-Gelman-Rubin diagnostic plots to ensure that the potential scale reduction factor (PSRF) came close to 1.0. Effect sizes were quantified as mean differences (MDs) with 95% CIs. Therapeutic efficacy hierarchy was determined via Surface Under the Cumulative Ranking Area (SUCRA) metrics, with higher values indicating superior performance (23). For network visualization, therapy comparison networks were constructed using STATA 15.0 (node size representing sample size, edge thickness reflecting comparison frequency), and the potential bias of publication was detected via Funnel plots. Cumulative ranking plots were generated using the ggplot2 package in R 4.3.0.

## Results

### Study selection process and results

**Figure 1:**
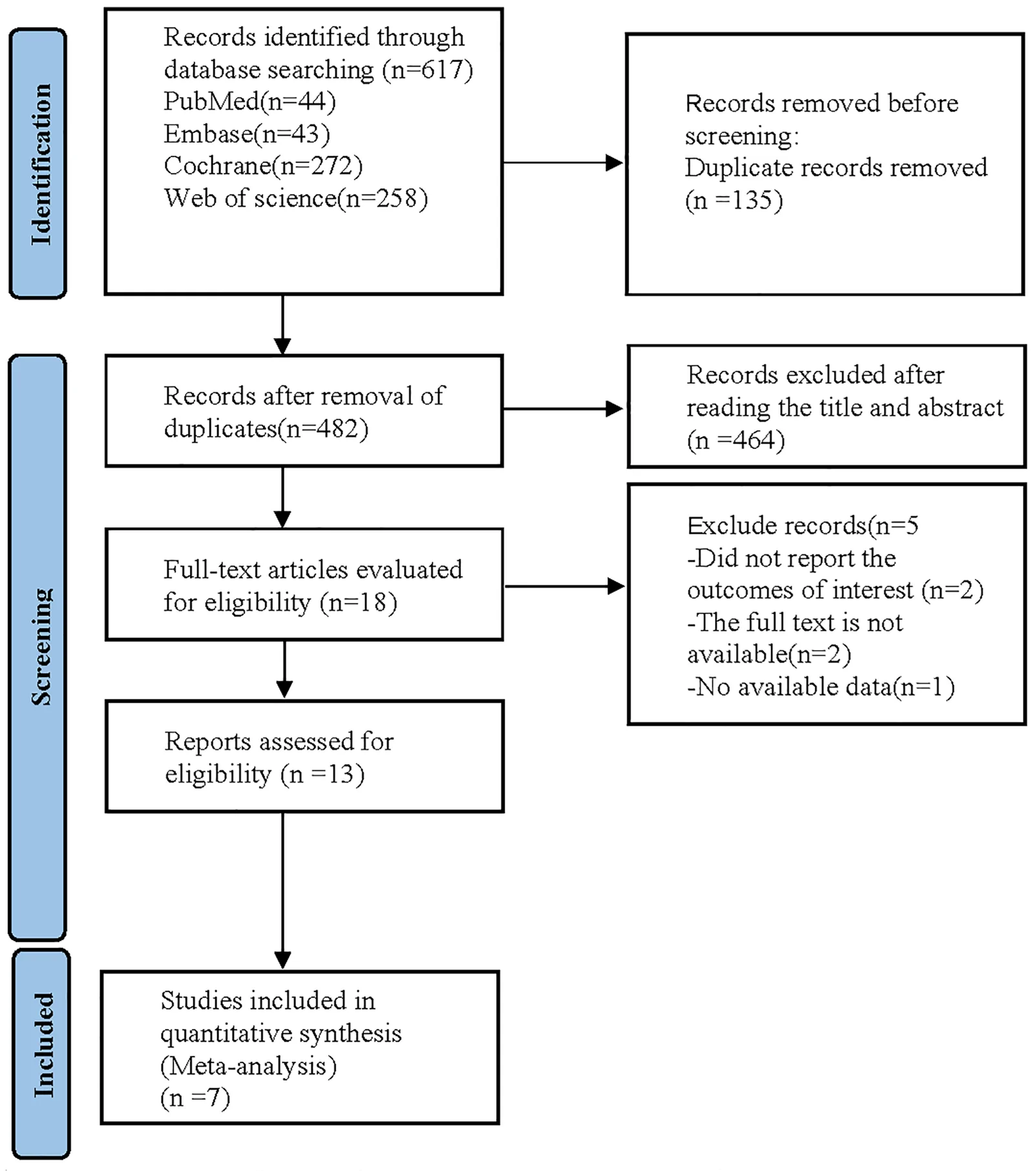
Flowchart.

Initial database searches identified 617 records. After removing 135 duplicates and excluding 464 irrelevant studies through title/abstract screening, 13 full-text articles underwent eligibility review. Ultimately, seven RCTs (10, 24–29) met inclusion criteria (**Figure 1**). For the Ro-CHOPLYSA trial, only the final analysis data (29) were included in the quantitative synthesis; the primary analysis (27) was not used as a separate data source to avoid duplication and to utilize the most mature follow-up data.

### Basic characteristics of included studies

A total of seven studies were included in the final analysis, encompassing 1,334 patients with PTCL, comprising 818 males and 516 females. The median age across studies was approximately 59 years. The first-line therapeutic regimens involved CHOP, VIP-rABVD, GEM-P, A+CHP, GDPT, Ro-CHOP, and ICED. The specific baseline characteristics of the studies included in the analysis are summarized in **Table 1**.

**Table 1:** Baseline characteristics of included studies.

Study	Year	Country	Sample size	Gender(M/F)	Mean age	ECOG performance	Intervention	Follow-up(month)	Outcome
Simon	2010	UK	VIP-rABVD:43 CHOP:43	56/30	VIP-rABVD:52 CHOP:49	0-4	VIP-rABVD: VIP cycles (1, 3 and 5) included etoposide 100 mg/m2 /d IV days 1–3, ifosfamide 1000 mg/m2 /d days 1–5 and cisplatin 20 mg/m2 /d as a continuous infusion on days 1–5. The three cycles of rABVD (cycles 2, 4 and 6) included on days 1 and, doxorubicin 50 mg/m2 /d, bleomycin 10 mg/m2 /d, vinblastine 10 mg/m2 /d and dacarbazine 375 mg/m2 /d. CHOP: cyclophosphamide 750 mg/m² IV Day1 doxorubicin 50 mg/m² IV Day1 vincristine 1·4 mg/m² [maximum 2 mg] IV day 1, prednisolone 100 mg po Day1-5	168	EFS,OS,ORR, DCR, AES
Gleeson	2018	UK	CHOP:43 GEM-P:44	62/25	CHOP:64 GEM-P:61	no	CHOP: cyclophosphamide 750 mg/m² IV Day1 doxorubicin 50 mg/m² IV Day1 vincristine 1·4 mg/m² [maximum 2 mg] IV day 1, prednisolone 100 mg po Day1-5 GEM-P: gemcitabine 1000 mg/m² IV Day 1, 8, 15 cisplatin 100 mg/m² IV Day 15, methylprednisolone 1000 mg IV or PO Day 1–5	no	OS, ORR, DCR, AES
Sun	2020	China	GDPT:77 CHOP:76	100/53	GDPT:52 CHOP:48.5	no	GDPT: gemcitabine 0.8g/m2 IV Day 1, 8 cisplatin 25mg/m2 IV Day1-3 prednisone 60mg/m2 PO d1–5,; thalidomide starting at 50mg, then increasing by 50– 200mg every day if there are few side effects, taken before going to bed until the end of the project. CHOP: cyclophosphamide 750 mg/m² IV Day1 doxorubicin 50 mg/m² IV Day1 vincristine 1·4 mg/m² [maximum 2 mg] IV day 1, prednisolone 60 mg po Day1-5	120	PFS, OS, ORR, DCR, AES
Horwitz	2019	International** (17 countries)**	A-CHP:226 CHOP:226	284/168	A_CHP:58 CHOP:58	0-2	A_CHP:brentuximab vedotin 1·8 mg/kg IV Day1_ cyclophosphamide 750 mg/m² IV Day1 doxorubicin 50 mg/m² IV Day1 prednisolone 100 mg po Day1-5 CHOP: cyclophosphamide 750 mg/m² IV Day1 doxorubicin 50 mg/m² IV Day1 vincristine 1·4 mg/m² [maximum 2 mg] IV day 1, prednisolone 100 mg po Day1-5	66	PFS, OS, ORR, DCR, AES
Bachy	2022	International (LYSA)	Ro-CHOP:211 CHOP:210	261/160	Ro-CHOP:65 CHOP:65	0-3	Ro-CHOP: cyclophosphamide 750 mg/m² IV Day1 doxorubicin 50 mg/m² IV Day1 vincristine 1·4 mg/m² [maximum 2 mg] IV day 1, prednisolone 60 mg po Day1-5 romidepsin 12 mg/m2 IV Day1, 8 CHOP: cyclophosphamide 750 mg/m² IV Day1 doxorubicin 50 mg/m² IV Day1 vincristine 1·4 mg/m² [maximum 2 mg] IV day 1, prednisolone 40 mg po Day1-5	78	PFS, OS, ORR, DCR, AES
Kim	2023	South Korea	CHOP:69 ICED:66	85/50	CHOP:57 ICED:57	0-2	CHOP: cyclophosphamide 750 mg/m² IV Day1 doxorubicin 50 mg/m² IV Day1 vincristine 1·4 mg/m² [maximum 2 mg] IV day 1, prednisolone 100 mg po Day1-5 ICED:ifosfamide 1, 670 mg/m2 IV days 1–3, carboplatin 5 x area under the curve (AUC) IV Day 1, etoposide 100 mg/m2 IV days 1–3, dexamethasone 40 mg IV or PO days 1–4	80	PFS, OS, ORR, DCR, AES
V. Camus, C	2024	International (LYSA)	Ro-CHOP:211 CHOP:210	261/160	Ro-CHOP:65 CHOP:65	0-3	Ro-CHOP: cyclophosphamide 750 mg/m² IV Day1 doxorubicin 50 mg/m² IV Day1 vincristine 1·4 mg/m² [maximum 2 mg] IV day 1, prednisolone 60 mg po Day1-5 romidepsin 12 mg/m2 IV Day1, 8 CHOP: cyclophosphamide 750 mg/m² IV Day1 doxorubicin 50 mg/m² IV Day1 vincristine 1·4 mg/m² [maximum 2 mg] IV day 1, prednisolone 40 mg po Day1-5	96	PFS, OS, ORR, DCR, AES

M/F, male/female; ECOG, performance, Eastern Cooperative Oncology Group performance; VIP- rABVD, Etoposide, ifosfamide, cisplatin alternate with doxorubicin, bleomycin, vinblastine, and dacarbazine; GEM-P: Gemcitabine Cisplatin Methylprednisolone; GDPT, gemcitabine, cisplatin, prednisone, thalidomide; A-CHP, brentuximab vedotin, cyclophosphamide, doxorubicin, prednisone; CHOP, cyclophosphamide, doxorubicin, vincristine, prednisone; ICED, Ifosfamide, carboplatin, etoposide, dexamethasone; Ro-CHOP, Romidisine, cyclophosphamide, doxorubicin, vincristine, prednisone; PFS, Progression-free Survial; OS, Overall Survival; ORR: Objective response rate; ORR, Objective Response Rate; DCR, Disease control rate; AES, Adverse event; DXM, dexamethasone; IV, intravenous; IM, intramuscular; PO, orally; RT, Radiotherapy.

For the Ro-CHOPLYSA trial, only the final analysis data (29) were included in the quantitative synthesis; the primary analysis (27) was not used as a separate data source to avoid duplication and to utilize the most mature follow-up data.

### Risk of bias assessment

**Figure 2:**
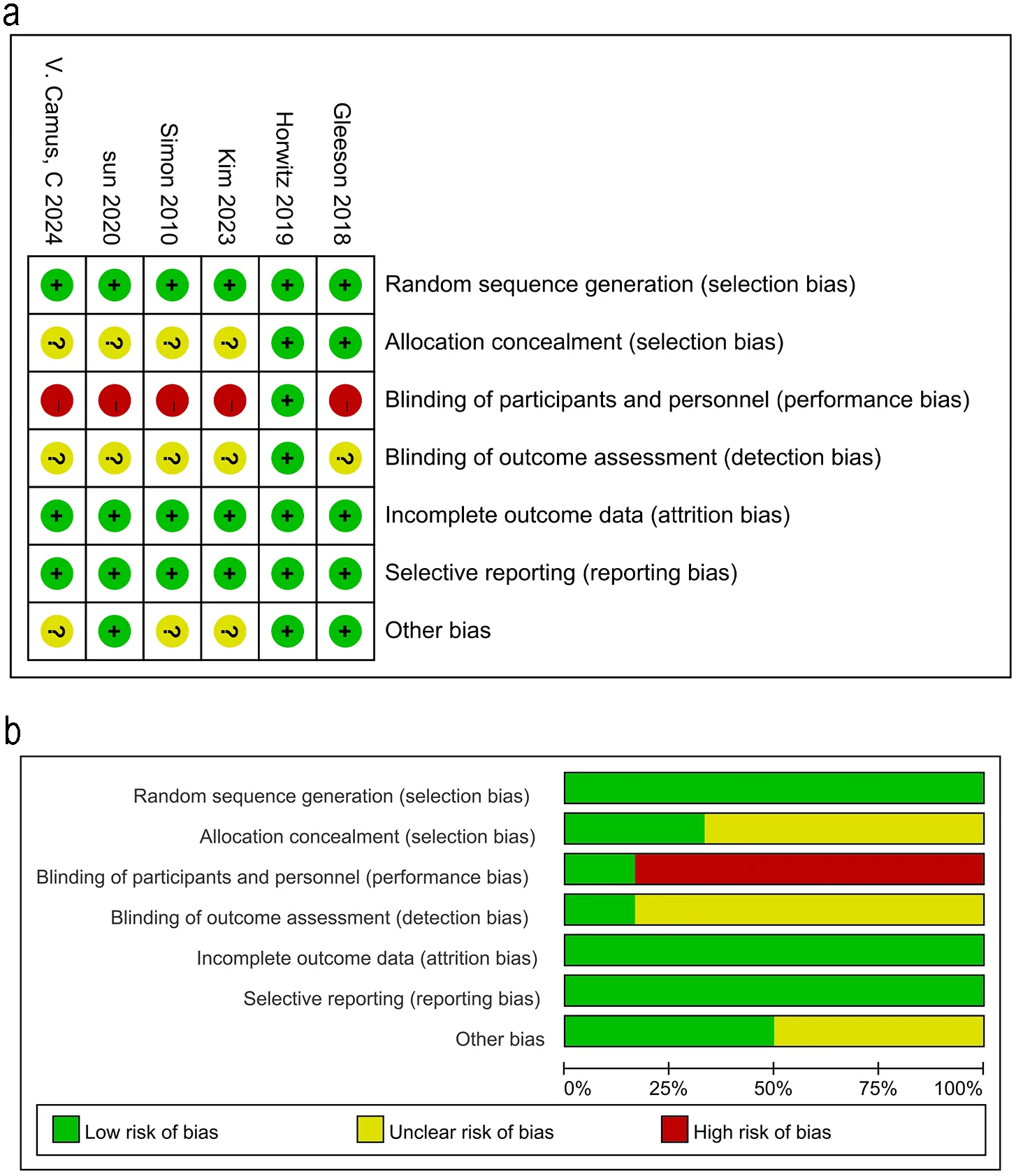
Figures of evidence synthesis and evaluation. **(A)** Risk of bias summary, **(B)** Risk of bias diagram.

All the studies included in the NMA explicitly documented their blinding procedures and demonstrated low risks concerning incomplete outcome data and selective reporting. No additional potential biases were identified during quality appraisal. Collectively, the analyzed trials exhibited minimal overall risk of bias. The detailed assessment of the risk of bias is presented in **Figure 2**.

### NMA results

#### OS

**Figure 3:**
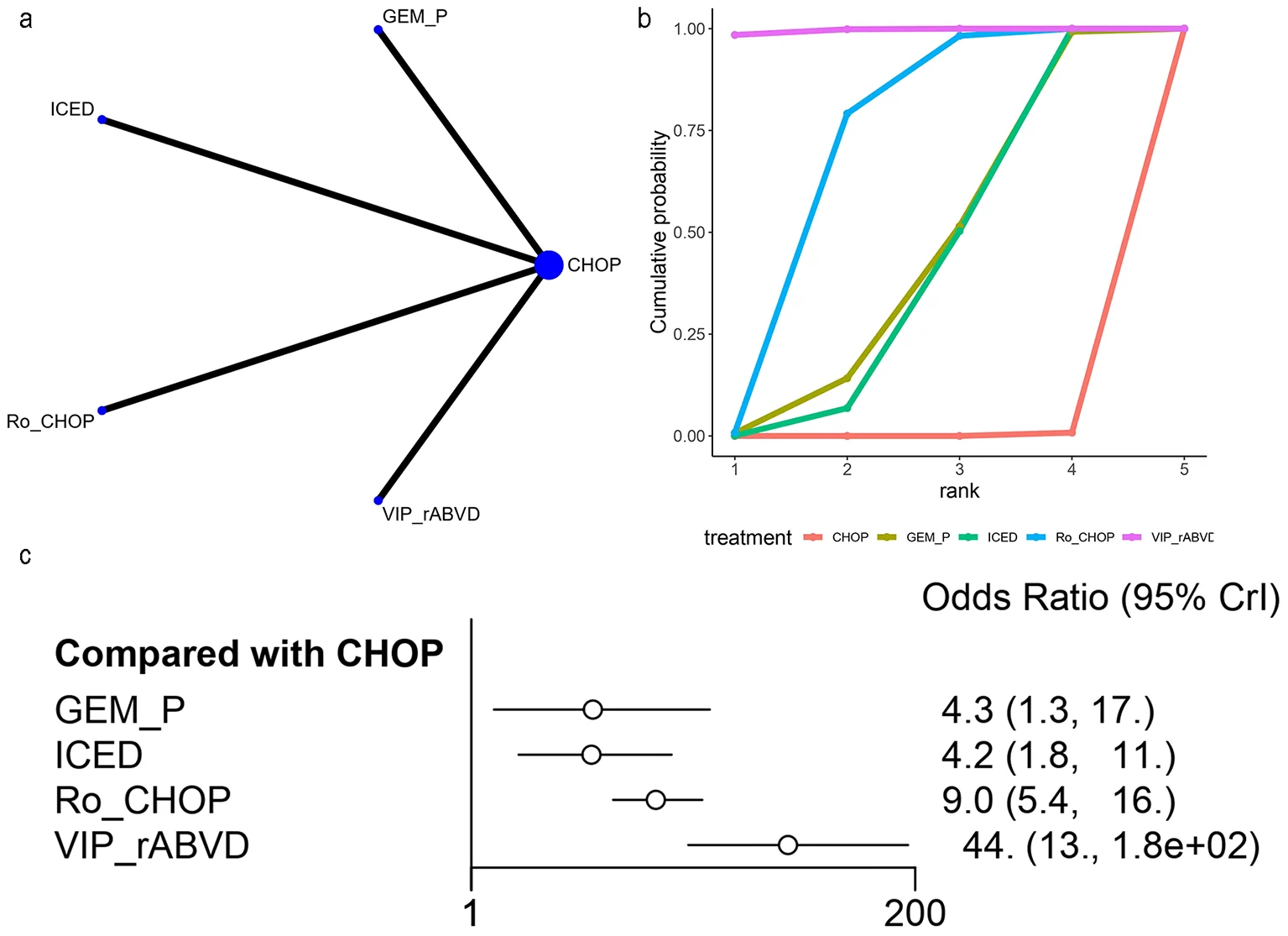
Figures of evidence synthesis and evaluation. **(A)** Network diagram, **(B)** Cumulative probability graph, **(C)** Forest Plot.

Seven studies (10, 24–29) reported OS outcomes (**Figure 3**). No closed loops were observed in the network diagram (**Figure 3A**), and Given the star-shaped network structure with CHOP at the center and no closed loops, formal inconsistency testing could not be performed. All comparisons between non-CHOP regimens are consequently indirect (GDPT vs. GEM-P vs. ICED vs. Ro-CHOP vs. VIP-rABVD vs. A-CHP vs. CHOP). Compared with conventional CHOP, A-CHP [HR = 0.67, 95% CI (0.15, 3.03)], GDPT [HR = 0.55, 95% CI (0.06, 5.00)], GEM-P [HR = 0.70, 95% CI (0.05, 11.10)], Ro-CHOP [HR = 0.88, 95% CI (0.33, 2.30)] exhibited potential improvement in OS (**Figure 3C**). Notably, GDPT exhibited superiority over A-CHP, GEM-P, and Ro-CHOP (see **Supplementary Table 1**). SUCRA was highest for GDPT (62.4%), followed by A-CHP (58.1%), GEM-P (55.4%), Ro-CHOP (47.2%), ICED (43.5%), CHOP (41.7%), and VIP-rABVD (41.7%) (**Figure 3B**; **Table 2**).

**Table 2:** Summary of findings (SoF) table.

Treatment	OS(SUCRA, %)	PFS(SUCRA, %)	NOS subgroup PFS(SUCRA ,%)	AITL subgroup PFS(SUCRA, %)	ORR(SUCRA, %)	DCR(SUCRA, %)	AEanemia ≥ grade 3 (SUCRA, %)	AEneutropenia ≥grade 3 (SUCRA,%)	AE thrombocytopenia ≥grade 3 (SUCRA, %)
A_CHP	58.5	55.8	52.0	37.7	88.1	83.3	33.6	37.9	NA
CHOP	40.1	38.5	42.0	41.4	37.8	30.0	9.1	35.3	2.0
GDPT	62.9	61.1	51.8	65.0	84.4	81.6	NA	NA	NA
GEM_P	54.5	57.5	NA	NA	16.4	20.1	35.5	15.0	41.4
ICED	43.5	41.4	52.4	39.0	76.7	21.1	96.0	64.4	39.3
Ro_CHOP	48.0	45.7	51.9	67.0	43.0	63.8	75.7	57.3	69.5
VIP_rABVD	43.1	NA	NA	NA	15.8	NA	NA	90.0	99.6

SUCRA values represent ranking probabilities, not absolute effect estimates. For absolute effect estimates (HRs, ORs), please refer to the forest plots and league tables in the **Supplementary Materials**.

#### PFS

**Figure 4:**
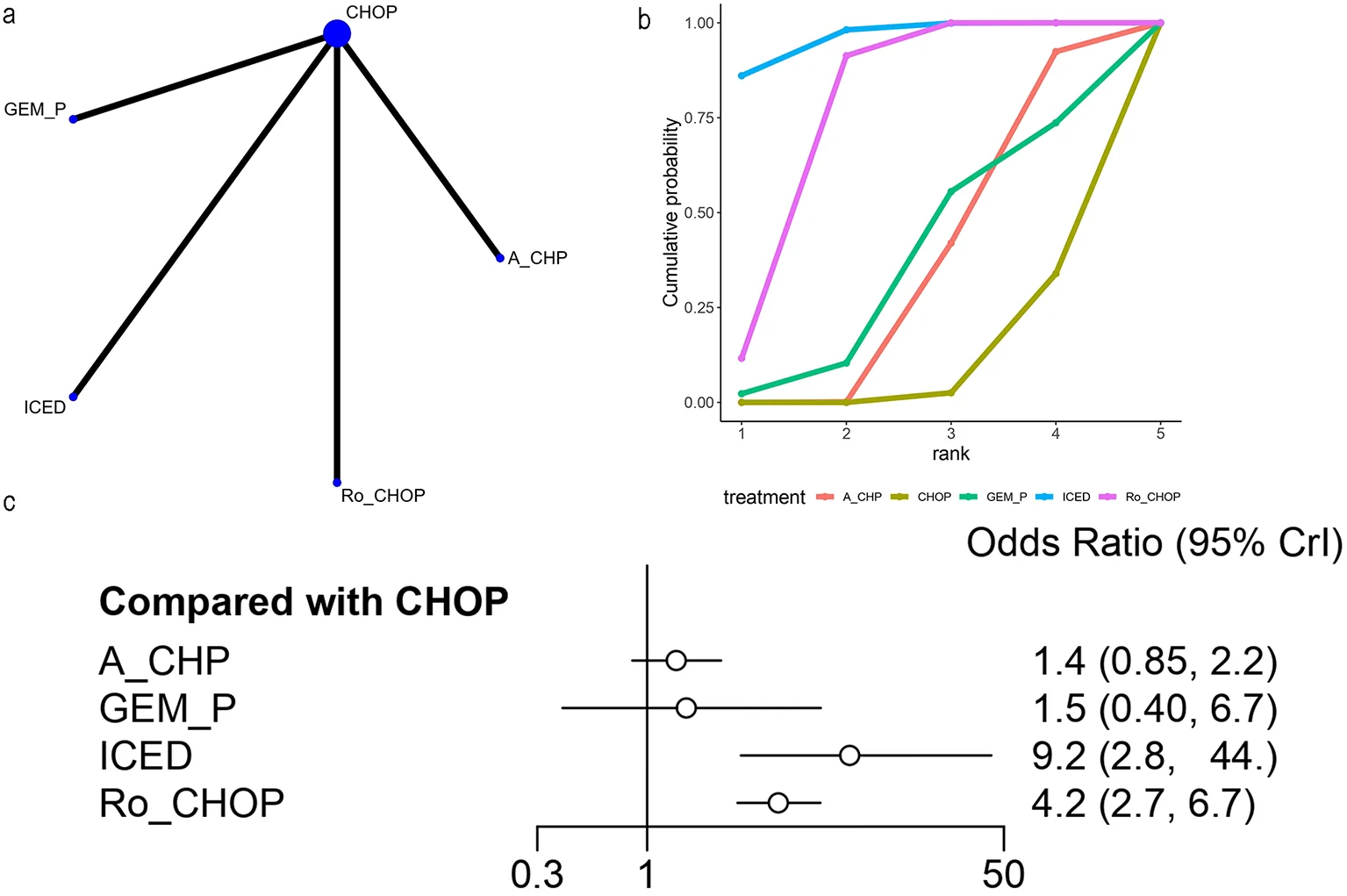
Figures of evidence synthesis and evaluation. **(A)** Network diagram, **(B)** Cumulative probability graph, **(C)** Forest Plot.

Six studies (10, 25–29) reported PFS outcomes (**Figure 4**). The network diagram did not show any closed loops (**Figure 4A**), and Given the star-shaped network structure with CHOP at the center and no closed loops, formal inconsistency testing could not be performed. All comparisons between non-CHOP regimens are consequently indirect (GDPT vs. GEM-P vs. ICED vs. Ro-CHOP v A-CHP vs.d CHOP. Compared with conventional CHOP, A-CHP [HR = 0.71, 95% CI (0.19, 2.70)], GDPT (HR = 0.57, 95% CI [0.07, 4.80)], GEM-P [HR = 0.56, 95% CI (0.015, 23.00)], and Ro-CHOP [HR = 0.89, 95% CI (0.22, 3.60)] exhibited a potential benefit in PFS (**Figure 4C**), with GDPT being more effective than GEM-P, A-CHP, and Ro-CHOP (see **Supplementary Table 2**). SUCRA was highest for GDPT (60.0%), followed by GEM-P (58.5%), A-CHP (53.3%), Ro-CHOP (49.5%), ICED (39.4%), and CHOP (37.2%) (**Figure 4B**; **Table 2**).

#### ORR

**Figure 5:**
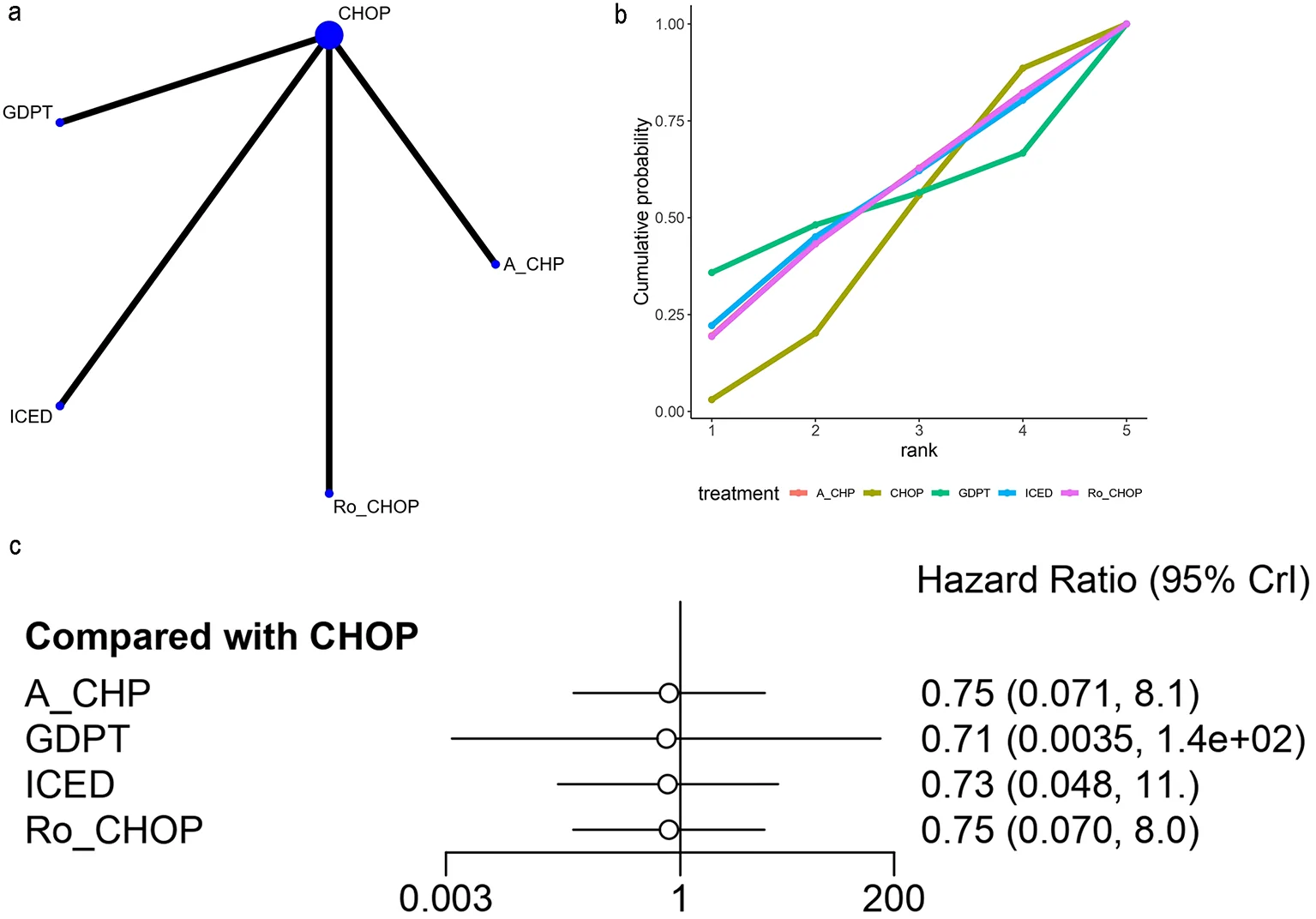
Figures of evidence synthesis and evaluation. **(A)** Network diagram, **(B)** Cumulative probability graph, **(C)** Forest Plot.

Seven studies (10, 24–29) reported ORR outcomes (**Figure 5**). An absence of closed loops was observed in the network diagram (**Figure 5A**), and Given the star-shaped network structure with CHOP at the center and no closed loops, formal inconsistency testing could not be performed. All comparisons between non-CHOP regimens are consequently indirect (GDPT vs. GEM-P vs. ICED vs. Ro-CHOP vs. VIP-rABVD vs A-CHP vs. CHOP. Compared with conventional CHOP, A-CHP [OR = 1.90, 95% CI (1.20, 3.10)], GDPT [OR = 2.00, 95% CI (1.00, 3.80)], Ro-CHOP [OR = 1.40, 95% CI (1.00, 1.80)], and VIP-rABVD [OR = 1.40, 95% CI (0.69, 1.60)] showed potential to improve ORR (**Figure 5B**), with A-CHP being superior to GDPT, Ro-CHOP, and VIP-rABVD (see **Supplementary Table 3**). SUCRA was highest for A-CHP (88.1%), followed by GDPT (84.4%), ICED (76.7%), Ro-CHOP (43.0%), CHOP (37.8%), GEM-P (16.4%), and VIP-rABVD (15.8%) (**Figure 5C**; **Table 2**).

#### DCR

**Figure 6:**
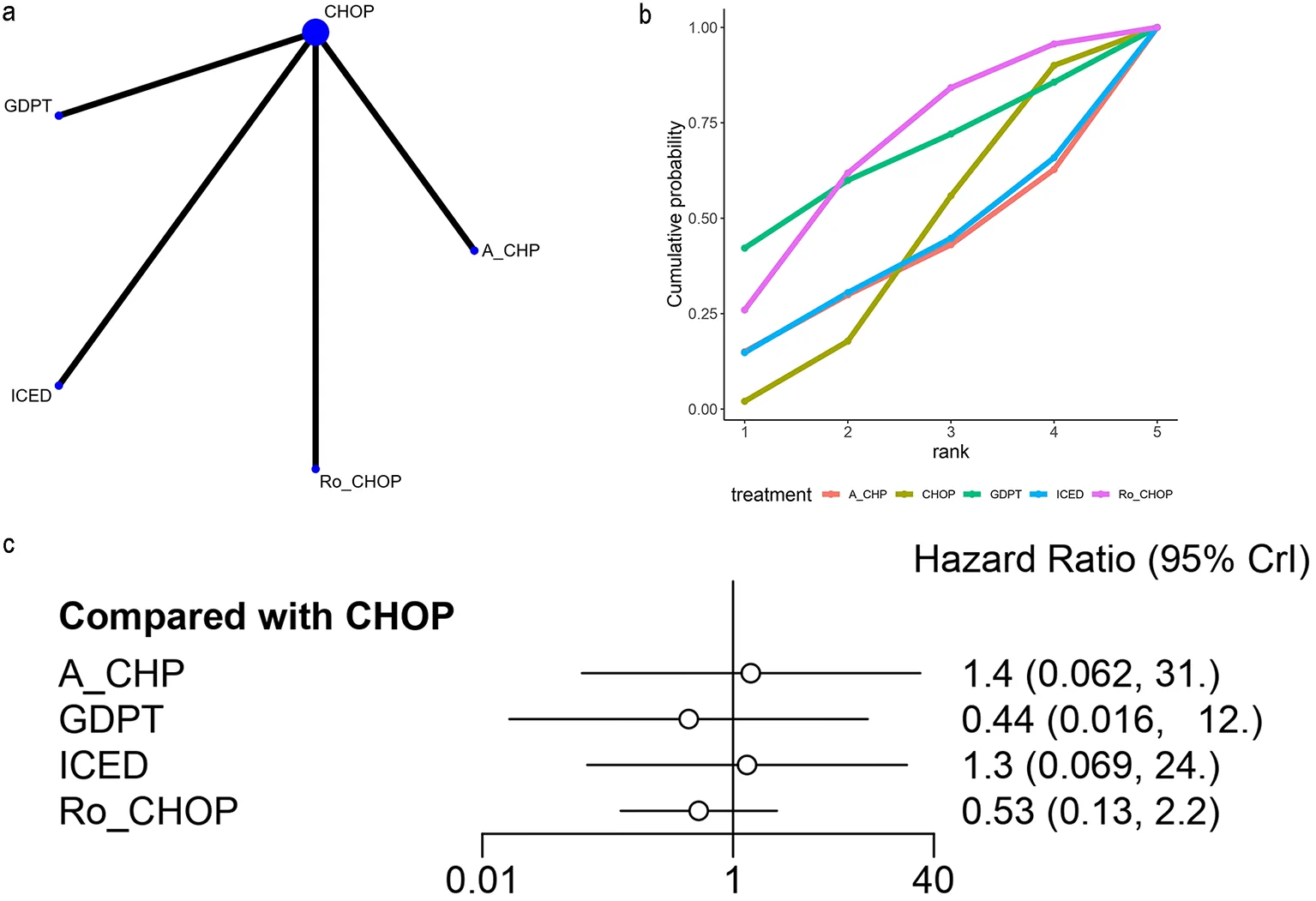
Figures of Evidence Synthesis and Evaluation. **(A)** Network diagram, **(B)** Cumulative probability graph, **(C)** Forest Plot.

Seven studies (10, 24–29) reported DCR outcomes (**Figure 6**). The network diagram exhibited no instances of closed loops (**Figure 6A**), and Given the star-shaped network structure with CHOP at the center and no closed loops, formal inconsistency testing could not be performed. All comparisons between non-CHOP regimens are consequently indirect (GDPT vs. GEM-P vs. ICED vs. Ro-CHOP vs. VIP-rABVD vs. A-CHP vs. CHOP). Compared with conventional CHOP, A-CHP [OR = 1.76, 95% CI (1.09, 2.88)], GDPT [OR = 1.79, 95% CI (0.93, 3.45)], and Ro-CHOP [OR = 1.40, 95% CI (1.00, 1.80)] demonstrated potential improvement in DCR (**Figure 6B**), with A-CHP outperforming GDPT and Ro-CHOP (see **Supplementary Table 4**). SUCRA was highest for A-CHP (83.3%), followed by GDPT (81.6%), Ro-CHOP (63.8%), CHOP (30.0%), GEM-P (20.1%), and VIP-rABVD (15.8%) (**Figure 6C**; **Table 2**).

#### Anemia (Grade ≥3)

**Figure 7:**
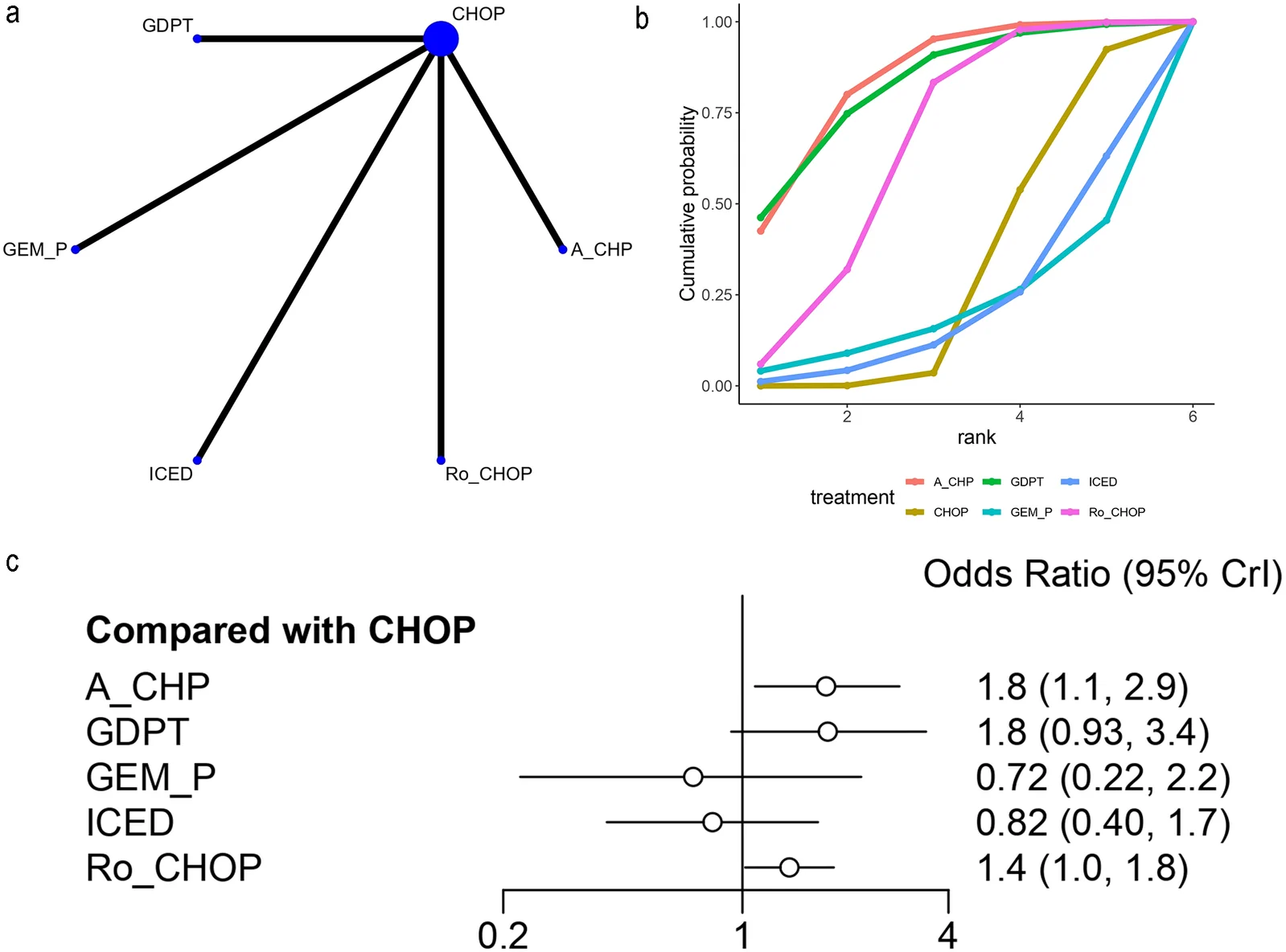
Figures of evidence synthesis and evaluation. **(A)** Network diagram, **(B)** Cumulative probability graph, **(C)** Forest Plot.

Four studies (10, 25, 27, 28) reported anemia (Grade ≥3) outcome (**Figure 7**). Analysis of the network diagram detected no closed loops (**Figure 7A**), and Given the star-shaped network structure with CHOP at the center and no closed loops, formal inconsistency testing could not be performed. All comparisons between non-CHOP regimens are consequently indirect (GEM-P vs. ICED vs. Ro-CHOP vs. A-CHP vs. CHOP). Compared with conventional CHOP, A-CHP [OR = 1.40, 95% CI (0.82, 2.20)], GEM-P [OR = 1.50, 95% CI (0.40, 6.70)], ICED [OR = 9.20, 95% CI (2.80, 44.00)], and Ro-CHOP [OR = 4.20, 95% CI (2.70, 6.70)] indicate an increased risk of Grade ≥3 anemia, especially with ICED and Ro-CHOP (**Figure 7B**). ICED posed the highest risk, followed by Ro-CHOP, GEM-P, A-CHP, and CHOP (see **Supplementary Table 5**). SUCRA was highest for ICED (96.0%), followed by Ro-CHOP (75.7%), GEM-P (35.5%), A-CHP (33.6%), and CHOP (9.1%) (**Figure 7C**; **Table 2**).

#### Thrombocytopenia (Grade ≥3)

**Figure 8:**
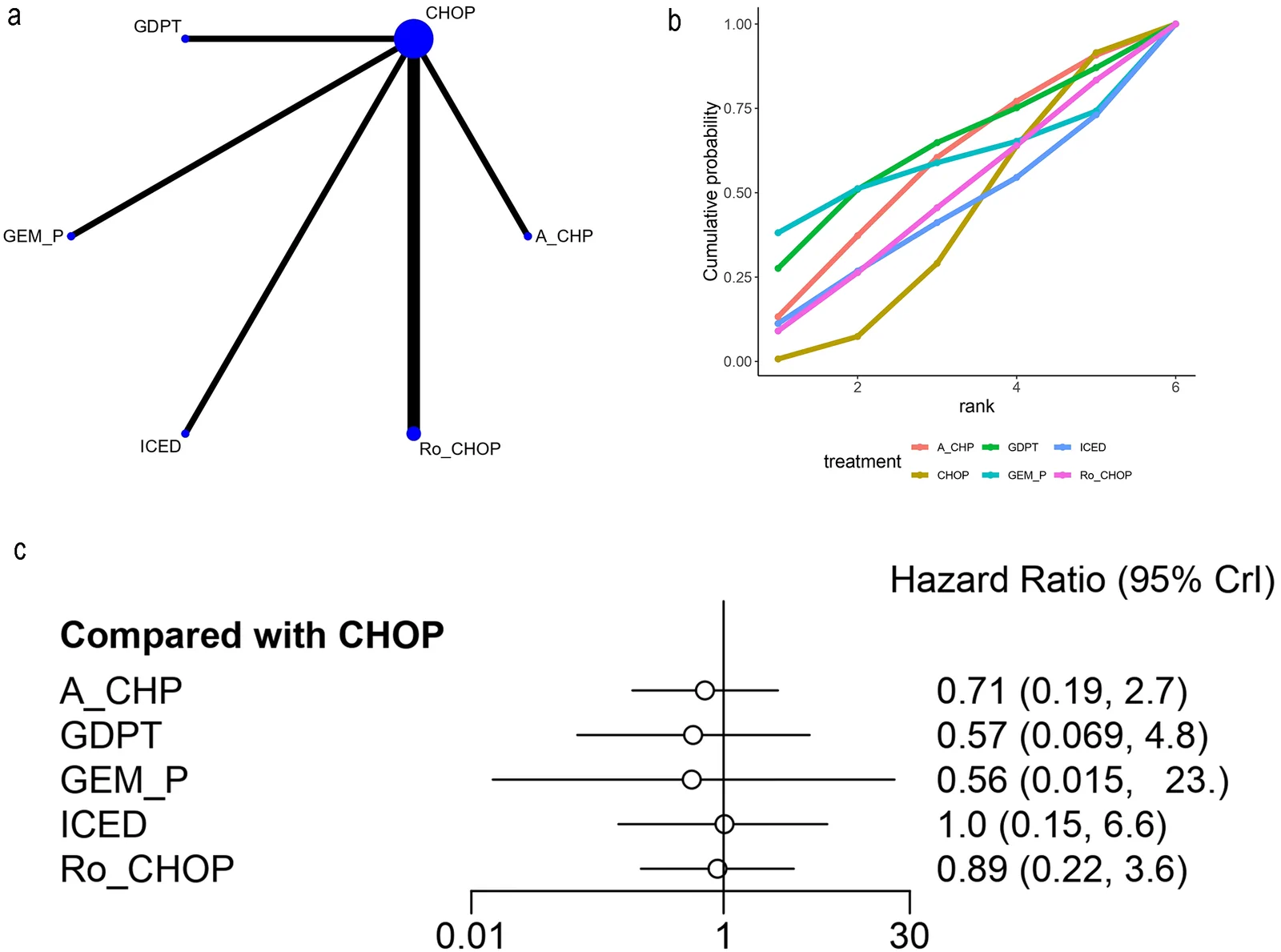
Figures of evidence synthesis and evaluation. **(A)** Network diagram, **(B)** Cumulative probability graph, **(C)** Forest Plot.

Four studies (10, 24, 27, 28) reported thrombocytopenia (grade ≥3) outcome. Closed loops were not revealed in the network diagram (**Figure 8A**), and Given the star-shaped network structure with CHOP at the center and no closed loops, formal inconsistency testing could not be performed. All comparisons between non-CHOP regimens are consequently indirect (GEM-P vs. ICED vs. Ro-CHOP vs. A-CHP vs. CHOP). Compared with conventional CHOP, the odds ratios (OR) for grade ≥3 thrombocytopenia were as follows: GEM-P, OR = 4.3, 95% CI (1.3, 17.0); ICED, OR = 4.2, 95% CI (1.3, 11.0); Ro-CHOP, OR = 9.0, 95% CI (5.4, 16.0); and VIP-rABVD, OR = 50, 95% CI (14.29, 100), suggesting an increased risk associated with these regimens (**Figure 8B**). Notably, VIP-rABVD posed the highest risk, exceeding that of Ro-CHOP, GEM-P, ICED, and CHOP (see **Supplementary Table 6**). SUCRA was highest for VIP-rABVD (96.0%), followed by Ro-CHOP (69.5%), GEM-P (41.4%), ICED (39.3%), and CHOP (2.0%) (**Figure 8C**; **Table 2**).

#### Neutropenia (Grade ≥3)

**Figure 9:**
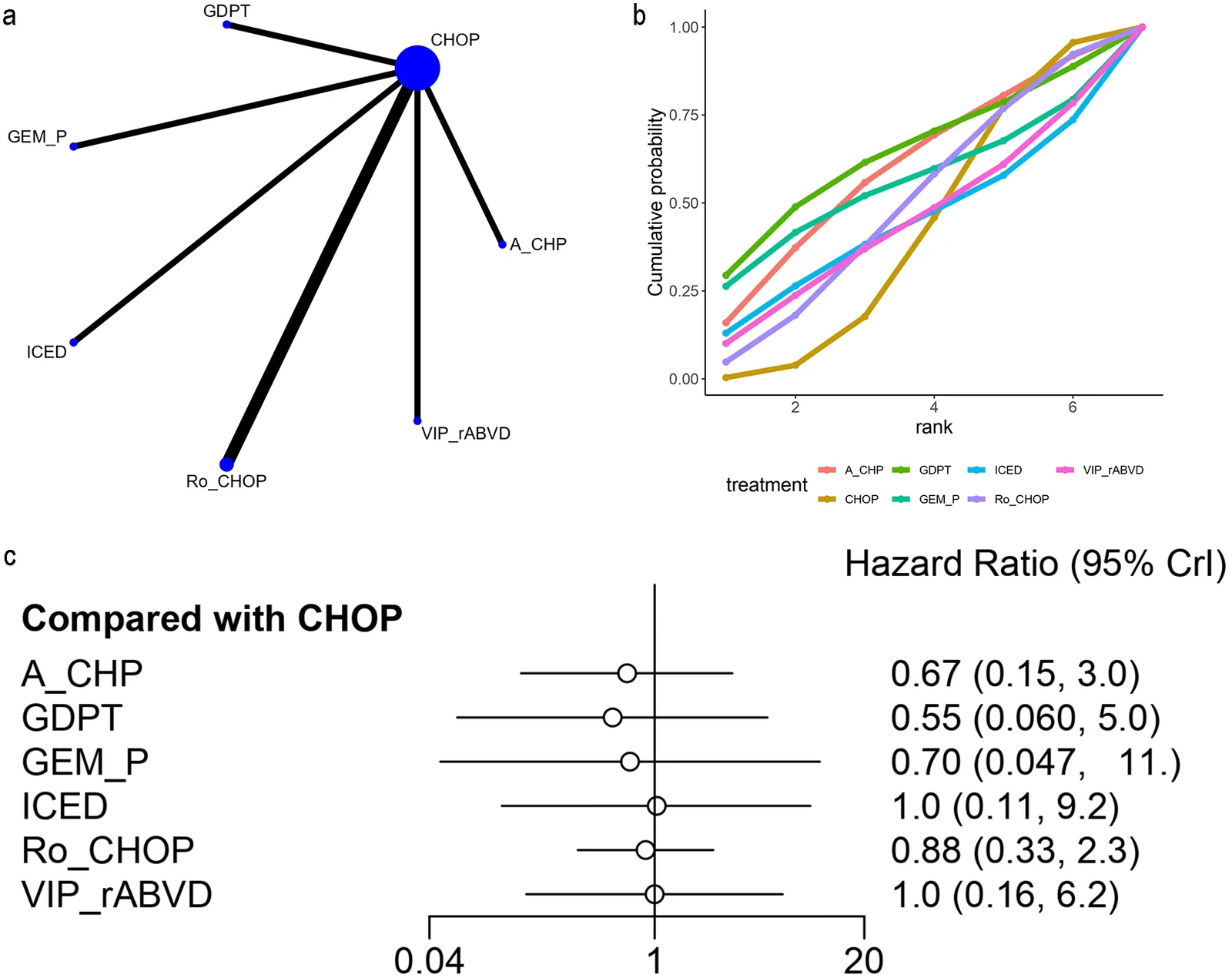
Figures of evidence synthesis and evaluation. **(A)** Network diagram, **(B)** Cumulative probability graph, **(C)** Forest Plot.

Five studies (10, 24–28) reported neutropenia (grade ≥3) outcome (**Figure 8**). The network diagram was free of closed loops (**Figure 9A**), and Given the star-shaped network structure with CHOP at the center and no closed loops, formal inconsistency testing could not be performed. All comparisons between non-CHOP regimens are consequently indirect (GEM-P vs. ICED vs. Ro-CHOP vs. VIP-rABVD vs.A-CHP vs. CHOP). Compared with conventional CHOP, the following ORs were observed for increased risk of grade ≥3 neutropenia: A-CHP, OR = 1.0, 95% CI (0.056, 18.0); ICED, OR = 2.7, 95% CI (0.14, 53.0); Ro-CHOP, OR = 2.0, 95% CI (0.11, 36.0); and VIP-rABVD, OR = 12.0, 95% CI (0.57, 280) (**Figure 9B**). Among these, VIP-rABVD posed the greatest risk, surpassing A-CHP, ICED, and Ro-CHOP (**Supplementary Table 7**). SUCRA was highest for VIP-rABVD (90.0%), followed by ICED (64.4%), Ro-CHOP (57.3%), A-CHP (37.9%), CHOP (35.3%), and GEM-P (15.0%) (**Figure 9C**; **Table 2**).

### PFS in AITL and PTCL-NOS subgroups

**Figure 10:**
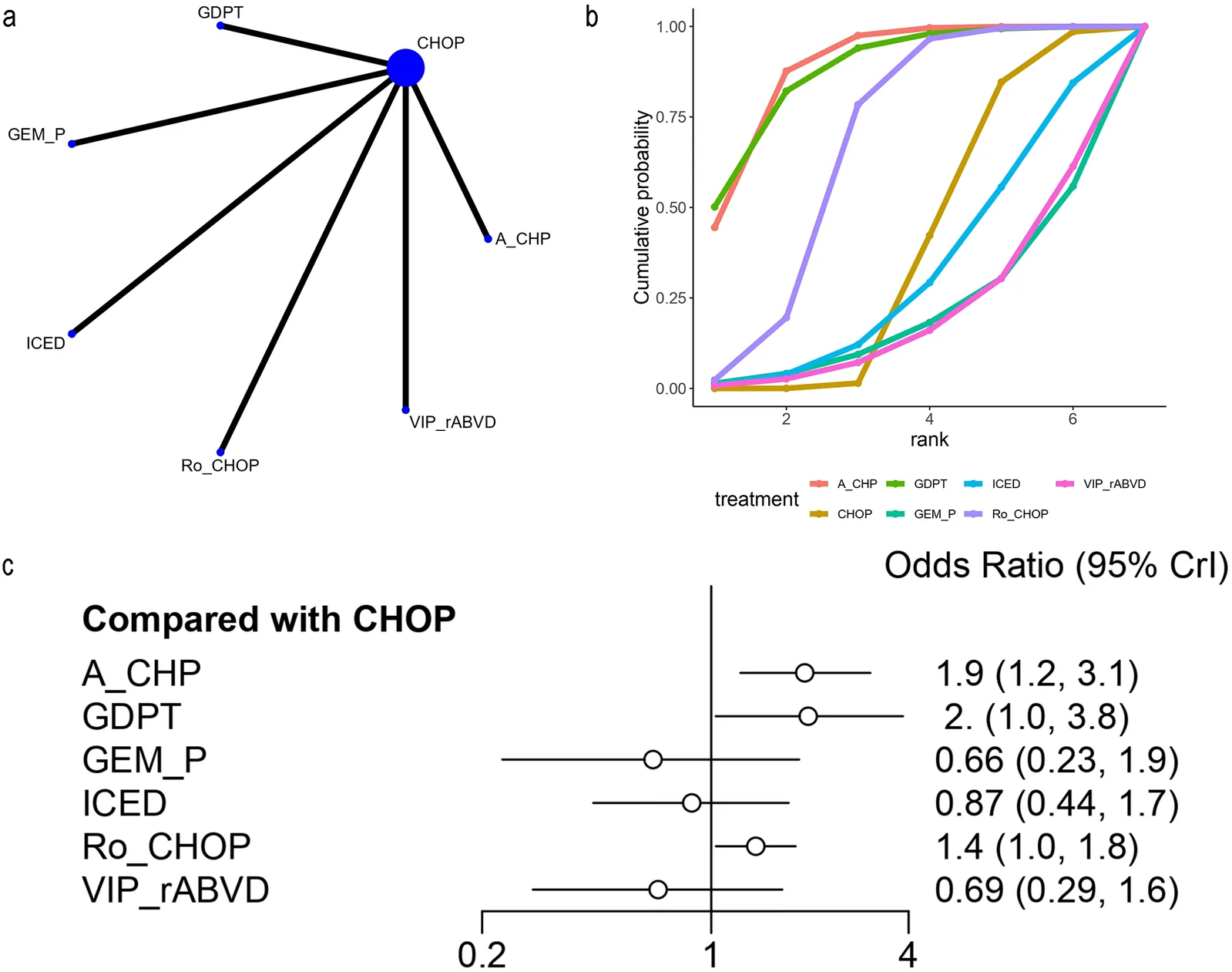
Figures of evidence synthesis and evaluation. **(A)** Network diagram, **(B)** Cumulative probability graph, **(C)** Forest Plot.

Four studies (25–28) reported PFS in AITL subgroup (**Figure 10**). No closed loops were present in the network diagram (**Figure 10A**), and Given the star-shaped network structure with CHOP at the center and no closed loops, formal inconsistency testing could not be performed. All comparisons between non-CHOP regimens are consequently indirect (GDPT vs. ICED vs. Ro-CHOP vs. A-CHP vs. CHOP). Compared with conventional CHOP, the following hazard ratios (HRs) were concluded: GDPT, HR = 0.44, 95% CI (0.016, 12.0); Ro-CHOP, HR = 0.58, 95% CI (0.13, 2.2), both indicating potential improvements in PFS (**Figure 10C**). Ro-CHOP ranked higher than GDPT (**Supplementary Table 8**). SUCRA was highest for Ro-CHOP (67.0%), followed by GDPT (65.0%), CHOP (41.4%), ICED (39.0%), and A-CHP (37.7%) (**Figure 10B**; **Table 2**).

**Figure 11:**
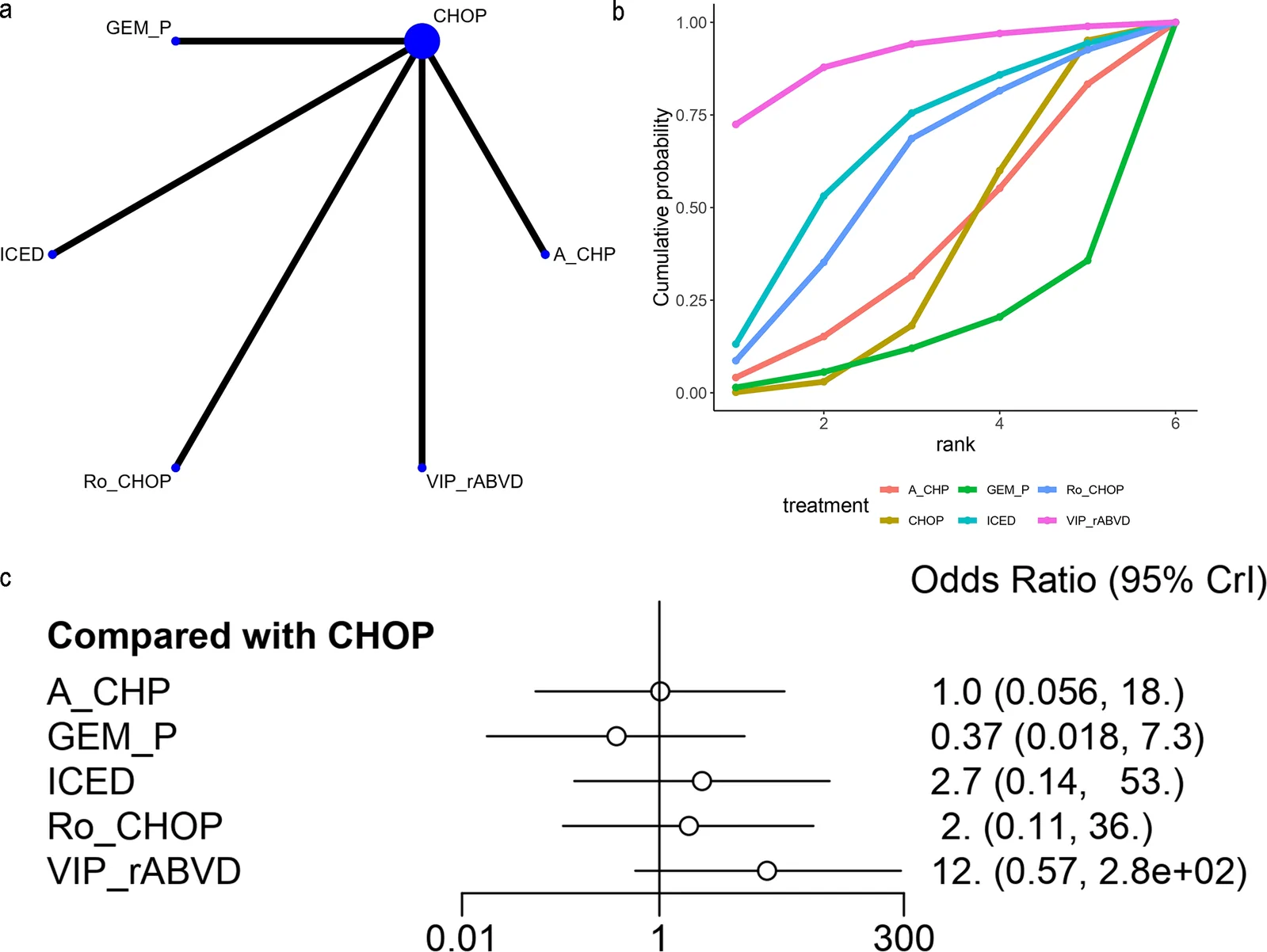
Figures of evidence synthesis and evaluation. **(A)** Network diagram, **(B)** Cumulative probability graph, **(C)** Forest Plot.

Four studies (25–28) reported PFS in PTCL-NOS subgroup (**Figure 11**). Again, No closed loops were found in the network diagram, and Given the star-shaped network structure with CHOP at the center and no closed loops, formal inconsistency testing could not be performed. All comparisons between non-CHOP regimens are consequently indirect (GDPT vs. ICED vs. Ro-CHOP vs. A-CHP vs. CHOP). Compared with conventional CHOP, the following HRs were reported: A-CHP, HR = 0.75, 95% CI (0.071, 8.1); GDPT, HR = 0.71, 95% CI (0.0035, 100); ICED, HR = 0.73, 95% CI (0.048, 11.0); and Ro-CHOP, HR = 0.75, 95% CI (0.07, 8.0) (**Figure 11B**). ICED demonstrated a slight advantage over the other regimens A-CHP, GDPT, ICED, and Ro-CHOP (**Supplementary Table 9**). SUCRA was highest for ICED (52.4%), followed by A-CHP (52.0%), Ro-CHOP (51.9%), GDPT (51.8%), and CHOP (42.0%) (**Figure 11C**; **Table 2**).

### Evaluation of publication bias

**Figure 12:**
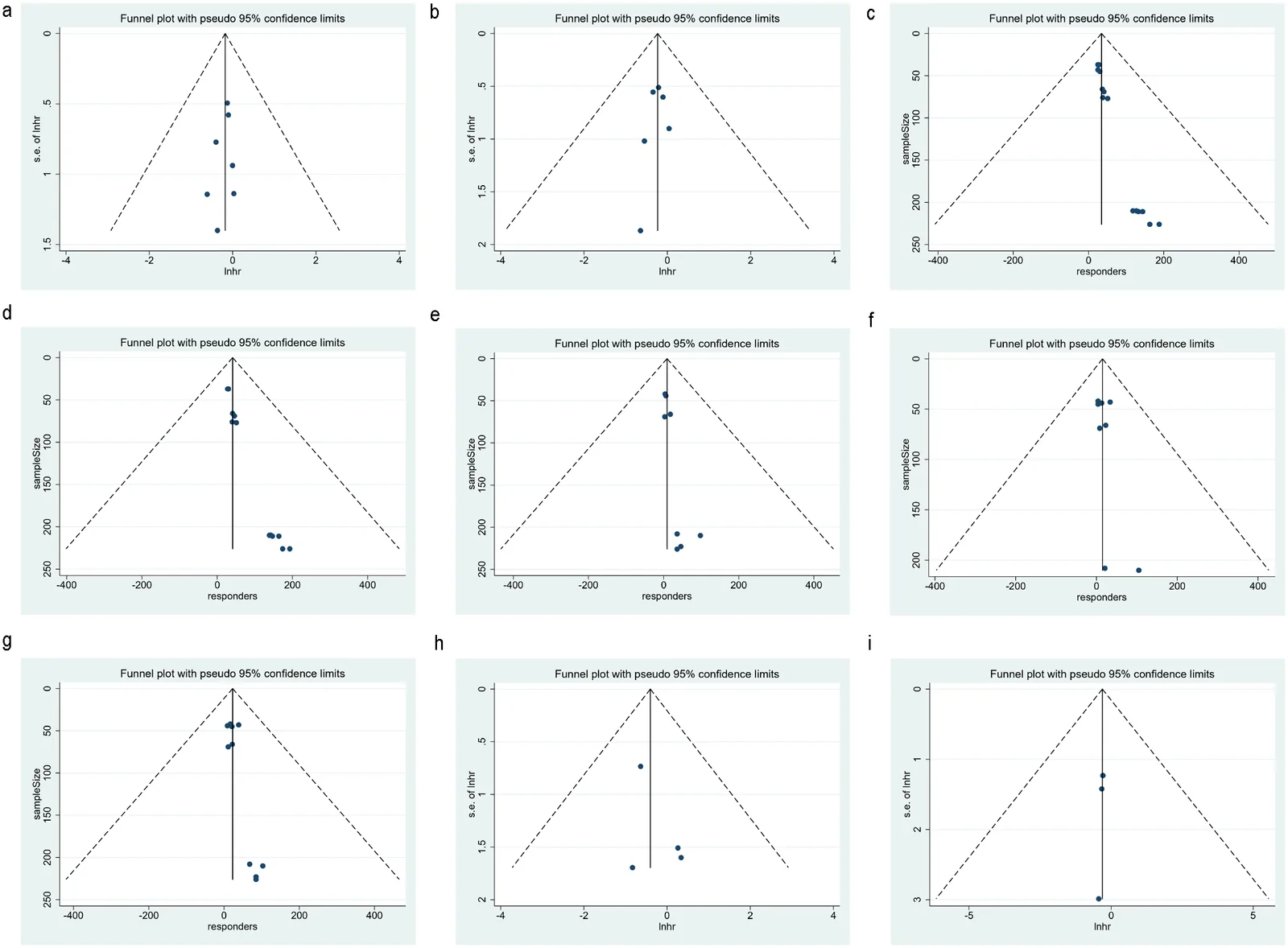
Figures of evidence synthesis and evaluation. **(A)** OS funnel plot, **(B)** PFS funnel plot, **(C)** ORR funnel plot, **(D)** DCR funnel plot, **(E)** AE anemia (≥ grade 3) funnel plot, **(F)** AE thrombocytopenia (grade ≥3) funnel plot, **(G)** AE neutropenia (grade ≥ 3) funnel plot, **(H)** PFS in AITL subgroup, **(I)** PTCL-NOS Subgroup PFS.

Funnel plots were applied in the assessment of the risk of publication bias for first-line therapeutic regimens across multiple outcomes, including OS, PFS, ORR, DCR, anemia (grade ≥3), thrombocytopenia (grade ≥3), neutropenia (grade ≥3), and PFS in both the AITL and PTCL-NOS subgroups. The symmetrical distribution in these plots suggests no significant publication bias (**Figure 12**).

## Discussion

In evaluating first-line therapeutic regimens for PTCL patients from an aspect of OS, the findings indicated that the GDPT regimen yielded the highest SUCRA values for OS and PFS, followed by A-CHP and GEM-P. These novel regimens (A-CHP, GDPT, GEM-P) demonstrated potential survival advantages over conventional CHOP, as evidenced by HRs <1, suggesting superiority. ICED and VIP-rABVD showed HRs approximating 1, indicating similar efficacy to CHOP. Ro-CHOP had an HR of 0.88, but with a confidence interval encompassing 1. This indicated a slight possible benefit that did not reach statistical significance (**Figures 3C**, **4C**). Previous studies have shown that gemcitabine-based combinations significantly prolong survival with manageable toxicity. For instance, the GemOx regimen (gemcitabine plus oxaliplatin) in relapsed/refractory PTCL achieved a median OS of 26.2 months (5), outperforming CHOP and aligning with the HR findings. GDPT’s superiority in SUCRA rankings in terms of OS and PFS may be attributed to its synergistic pharmacological interactions: gemcitabine-induced DNA damage and cisplatin-induced crosslinking act synergistically, while dexamethasone enhances chemosensitivity by inhibiting the NF-κB pathway—mechanisms validated by pharmacokinetic data from Iqbal et al (30). This is consistent with Swerdlow SH et al., who reported a 58% ORR for gemcitabine-platinum combinations in relapsed/refractory PTCL (31). Additionally, a retrospective study (n=116) showed that gemcitabine-based regimens achieved a 3-year OS rate of 56.3% in treatment-naïve PTCL, significantly outperforming CHOP-based therapies (37.0%–47.0%) (32). The clinical benefit of A-CHP likely stems from brentuximab vedotin’s targeted mechanism against CD30+ tumor cells. As an antibody-drug conjugate (ADC), brentuximab vedotin binds specifically to CD30, internalizes into tumor cells, and releases MMAE, a microtubule-disrupting agent. MMAE can trigger cell cycle arrest and apoptosis. Additionally, MMAE exerts a bystander effect, eliminating nearby tumor cells (33, 34). In patients with relapsed/refractory CD30+ PTCL, single-agent brentuximab vedotin has demonstrated remarkable efficacy (35, 36). Phase I data from Michelle A et al. showed durable survival benefits with A-CHP in newly diagnosed CD30+ PTCL, with 50% of patients progression-free at 5 years—substantially better than CHOP, which offers only 20–40% 5-year PFS (37). However, the efficacy of A-CHP may be overestimated due to its evidence base being limited to CD30-positive cohorts. Moreover, heterogeneity in CD30 testing methods may introduce measurement bias. Notably, Ro-CHOP, which combines romidepsin with CHOP, exhibited a median HR of 0.88, suggesting potential survival benefits. However, the wide confidence interval (0.33–2.3) warrants cautious interpretation. This aligns with previous prospective phase II trials by Pellegrini C et al., which reported variable response rates to single-agent HDAC inhibitors in relapsed PTCL, suggesting a possible role in molecularly defined subtypes (38). Overall, novel regimens such as GDPT, A-CHP, and GEM-P appear to provide superior first-line survival outcomes in PTCL compared with traditional CHOP. Among them, GDPT showed the most favorable OS and PFS ranking, while A-CHP may offer curative potential in CD30-positive patients, albeit with efficacy estimates potentially affected by testing heterogeneity.

In evaluating the ORR and DCRof first-line therapeutic regimens in treatment-naïve PTCL patients, A-CHP (brentuximab vedotin plus chemotherapy) demonstrated significant superiority. Specifically, the ORR for A-CHP yielded an odds ratio (OR) of 1.90 (95% CI: 1.20–3.10), and GDPT showed an OR of 2.00 (95% CI: 1.00–3.80) (**Figure 5B**). For DCR, A-CHP exhibited an OR of 1.76 (95% CI: 1.09–2.88), and Ro-CHOP showed an OR of 1.40 (95% CI: 1.00–1.80) (**Figure 6B**). These results underscore the statistically significant advantage (p < 0.05) of the A-CHP regimen, with SUCRA values for ORR and DCR of 88.1% and 83.3%, respectively. This aligns with key clinical findings in CD30-positive PTCL patients. A multicenter phase I study reported an ORR of 100% and a complete response (CR) rate of 92% in patients with high CD30 expression (≥10%) treated with A-CHP (37). Even as monotherapy in relapsed/refractory PTCL, BV demonstrated substantial activity (ORR 41%) (36), supporting its synergistic efficacy in combination regimens. The GDPT regimen (gemcitabine, cisplatin, and thalidomide) also showed improved ORR (OR = 2.00), consistent with previous findings. In relapsed/refractory PTCL, GDPT achieved an ORR of 69% and a DCR of 82.8%, with manageable toxicity (37); Furthermore, a single-center, open-label study in China demonstrated enhanced activity using GDP combined with methotrexate (MTX) and PEG-asparaginase (PEG-L) in PTCL treatment. Mechanistically, gemcitabine enhances the cytotoxicity of cisplatin, while thalidomide inhibits the VEGF signaling pathway. The observed DCR benefit of Ro-CHOP (romidepsin plus CHOP) may be attributed to an increased rate of stable disease (SD). Romidepsin, a histone deacetylase (HDAC) inhibitor, has shown acceptable safety and a DCR of 52% when combined with vincristine and cyclophosphamide in relapsed/refractory neuroblastoma, suggesting possible synergy with other chemotherapeutics (34). It restores tumor suppressor gene expression and sensitizes tumor cells to chemotherapy via HDAC inhibition (39). Nonetheless, the observed increase in ORR did not reach statistical significance (95% CI: 1.0–1.8), indicating limited depth of response. In summary, the clinical and biological advantages of A-CHP and GDPT are supported by current evidence. While Ro-CHOP enhances DCR through epigenetic modulation, its ORR improvement is limited, highlighting the need for optimization of targeted strategies.

In assessing the safety of first-line regimens in treatment-naïve PTCL patients, this study focused on the top three most frequently reported grade ≥3 hematologic toxicities—anemia, thrombocytopenia, and neutropenia—due to their significant impact on treatment tolerability and clinical decision-making (e.g., transfusion or growth factor use), as well as their high incidence and complete data availability in T-cell lymphomas, minimizing reporting bias. In the present meta-analysis, A-CHP showed no statistically significant differences compared to CHOP in terms of grade ≥3 anemia (OR = 1.40; 95% CI: 0.82–2.20) or grade ≥3 neutropenia (OR = 1.00; 95% CI: 0.056–18.0), suggesting that hematologic toxicities associated with A-CHP are manageable. A prior multicenter phase II trial also demonstrated that the hematologic toxicity profile of BV combined with CHP was not increased and was even lower than that of CHOP (36). Pharmacologically, Brentuximab Vedotin is an anti-CD30 antibody-drug conjugate, and CD30 is minimally expressed in normal tissues. By specifically targeting CD30-positive tumor cells and inducing apoptosis, BV reduces off-target cytotoxic effects on normal hematopoietic cells (36, 40), consistent with the safety findings. It is important to note that this regimen primarily targets CD30-positive PTCL subtypes (e.g., ALCL), and safety data for CD30-negative patients remain limited, which may affect the generalizability of the safety assessment. Previous studies have reported a significantly increased risk of grade ≥3 thrombocytopenia with Ro-CHOP compared to CHOP (75% vs. 10.1%) (37), a trend corroborated by our meta-analysis (OR = 9.0; 95% CI: 5.4–16.0). Romidepsin, as an HDAC inhibitor, induces apoptosis via epigenetic regulation. However, HDAC inhibition may impair DNA repair in hematopoietic stem cells and, in combination with the myelotoxicity of CHOP (e.g., cyclophosphamide), may exacerbate thrombocytopenia and neutropenia (34). Although romidepsin may enhance antitumor activity through immune modulation, its hematologic risks—particularly thrombocytopenia—warrant careful risk-benefit assessment and appropriate management strategies. As for the GDPT regimen, adverse event data from included RCTs only reported general bone marrow suppression without specific grading for grade ≥3 hematologic toxicity; thus, detailed analysis was not conducted. Nevertheless, prior studies have shown that while GDPT is effective in relapsed/refractory PTCL, it also carries considerable hematologic toxicity, with grade 3–4 neutropenia occurring in approximately 50% of patients (37). Thalidomide’s anti-angiogenic properties may inhibit VEGF within the bone marrow microenvironment, thereby reducing platelet production (40). The potential synergy between chemotherapy and immunomodulation may improve efficacy, and several studies support the use of GDPT in frontline settings. However, this benefit comes at the cost of substantial myelosuppression. The current meta-analysis lacks sufficient data on grade ≥3 hematologic toxicities for GDPT, underscoring the need for further prospective studies to validate its safety. Other safety data revealed significantly higher hematologic toxicity risks with ICED, VIP-rABVD, and similar regimens compared to CHOP. The extreme hematologic risk observed in some cases may be attributed to rare events in small sample sizes, as indicated by the wide confidence intervals, suggesting that these results should be interpreted cautiously and validated in larger cohorts.

To address the considerable biological heterogeneity of PTCL (encompassing over 30 subtypes as defined by the WHO classification) and the variability in subtype composition across the included RCTs, the NMA investigating the first-line therapies for PTCLs utilized a random-effects model with complementary sensitivity analyses to account for inter-study heterogeneity. Given the differential treatment responses driven by subtype-specific biological mechanisms, we further conducted an exploratory analysis focusing on the two predominant subtypes included in the RCTs: AITL and PTCL-NOS. The results demonstrated marked heterogeneity across subtypes. In AITL, the Ro-CHOP regimen yielded a point estimate of hazard ratio (HR) of 0.58 (95% CI: 0.13–2.20), suggesting a potential therapeutic benefit. Although the confidence interval crossed the null value 1, this finding aligns with the observed sensitivity of AITL to anti-CD30 monoclonal antibodies in a prospective clinical trial by Sibon D et al. The biologic plausibility of the findings is supported by the surface under SUCRA probabilities: Ro-CHOP (67.0%) and GDPT (65.0%) ranked favorably, likely attributable to the high CD30 positivity rate (60–80%) and CXCL13 expression characteristic of the AITL microenvironment. Conversely, the lower SUCRA value for A-CHP (37.7%) may reflect the limited efficacy of histone deacetylase inhibitors in TFH-like phenotypes (40). In contrast, efficacy among therapeutic regimens for PTCL-NOS appeared more convergent (SUCRA ranging from 52.4% to 42.0%), highlighting the influence of its molecular heterogeneity on therapeutic outcomes. The marginal advantage of ICED (SUCRA 52.4%) may stem from its dose-intensified strategy conferring better control over aggressive variants (41). These findings suggest that current evidence remains insufficient to establish a definitive optimal regimen, consistent with the lack of validated therapeutic targets in PTCL-NOS (42). To sum up, while Ro-CHOP may improve PFS in AITL (SUCRA 67.0%), its wide confidence interval (HR 0.13–2.20) warrants cautious interpretation. The observed convergence in treatment efficacy in PTCL-NOS (SUCRA 52.4%–42.0%) underscores the need for prospective validation and molecular stratification in future studies.

## Limitations of this study

First, due to the rarity of PTCL patients, there are very few published Phase III RCTs. To synthesize the most comprehensive evidence currently available, we also included Phase II RCTs, which has affected the level of evidence to some extent. Second, although we rigorously screened the RCTs, there were differences in the composition of PTCL subtypes included in the individual studies. This clinical heterogeneity may have a potential impact on the pooled effect size, leading to some bias in the results. Third, the exclusion of non-hematologic risks from the toxicity analysis may underestimate the overall safety burden, and the wide confidence intervals suggest that the results should be interpreted with caution. Fourth, the review protocol was not prospectively registered on PROSPERO before data extraction, which may increase the risk of unplanned *post-hoc* outcome adjustments. To mitigate this limitation, the full pre-specified protocol with all planned analytical approaches has been deposited on Zenodo for open access, allowing independent verification of all pre-defined methods. Fifth, a methodological limitation of our network meta-analysis should be noted: all included trials compared investigational regimens against CHOP, and no head-to-head randomized comparisons exist between any two non-CHOP regimens. Consequently, the network structure for every outcome was a ‘star’ with CHOP at the center and no closed loops. In the absence of closed loops, formal inconsistency assessments cannot be performed. Therefore, all comparisons between non-CHOP regimens are indirect and should be interpreted with appropriate caution. Our SUCRA rankings should be viewed as hypothesis-generating rather than confirmatory evidence of comparative effectiveness between non-CHOP regimens.

## Conclusion

The A+CHP regimen offers a definite survival benefit for patients with peripheral T-cell lymphoma (PTCL), with manageable overall hematologic toxicity; the GDPT regimen has a higher objective response rate, but clinicians must be particularly vigilant regarding the risk of bone marrow suppression during clinical use. Although the Ro-CHOP regimen demonstrates acceptable disease control, the depth of remission is relatively insufficient, and bone marrow toxicity is particularly pronounced; subgroup analysis suggests that this regimen may prolong progression-free survival in patients with angioimmunoblastic T-cell lymphoma (AITL).PTCL of unspecified type (PTCL-NOS) exhibits significant molecular heterogeneity, and there are only slight differences in the efficacy of various treatment regimens. Based on the available clinical evidence, the A+CHP and GDPT regimens demonstrate clear advantages in terms of efficacy and safety and should be considered the preferred treatment options in clinical practice.

## Data Availability

The datasets presented in this study can be found in online repositories. The names of the repository/repositories and accession number(s) can be found in the article/**Supplementary Material**.
