# Supplementary material for: Evaluation of the efficacy and safety of first-line therapies for peripheral T-cell lymphoma: a systematic review and network meta-analysis
Source: Front Oncol. 2026 Aug 24;16:1912842. doi: 10.3389/fonc.2026.1912842 (PMC13547012; doi:10.3389/fonc.2026.1912842)
Supplement: Supplementary file 1 [file Table1.docx]

Table S1 OS League Table

| HR 95%cl |  |  |  |  |  |  |
| --- | --- | --- | --- | --- | --- | --- |
| A_CHP |  |  |  |  |  |  |
| 0.67 (0.15, 3.03) | CHOP |  |  |  |  |  |
| 1.23 (0.08, 17.97) | 1.82 (0.2, 16.8) | GDPT |  |  |  |  |
| 0.96 (0.04, 21.42) | 1.42 (0.09, 21.39) | 0.79 (0.02, 25.82) | GEM_P |  |  |  |
| 0.66 (0.05, 9.55) | 0.97 (0.11, 8.87) | 0.53 (0.02, 12.21) | 0.68 (0.02, 22.31) | ICED |  |  |
| 0.77 (0.13, 4.58) | 1.14 (0.43, 3) | 0.62 (0.06, 6.95) | 0.8 (0.04, 14.26) | 1.17 (0.11, 12.86) | Ro_CHOP |  |
| 0.68 (0.06, 7.15) | 1 (0.16, 6.25) | 0.55 (0.03, 9.81) | 0.7 (0.03, 18.9) | 1.03 (0.06, 18.09) | 0.88 (0.11, 7) | VIP_rABVD |

Table S2 PFS League Table

| HR 95%CL | | | | | |
| --- | --- | --- | --- | --- | --- |
| A_CHP |  |  |  |  |  |
| 0.71 (0.19, 2.7) | CHOP |  |  |  |  |
| 1.25 (0.1, 15.16) | 1.74 (0.21, 14.43) | GDPT |  |  |  |
| 1.28 (0.03, 61.05) | 1.79 (0.04, 67.57) | 1.03 (0.01, 70.78) | GEM_P |  |  |
| 0.7 (0.07, 7.27) | 0.98 (0.15, 6.82) | 0.57 (0.03, 9.6) | 0.56 (0.01, 34.57) | ICED |  |
| 0.8 (0.12, 5.49) | 1.12 (0.28, 4.52) | 0.64 (0.05, 7.99) | 0.63 (0.01, 31.3) | 1.14 (0.1, 11.94) | Ro_CHOP |

Table S3 PTCL-NOS Subgroup PFS League Table

| HR 95%cl | | | | |
| --- | --- | --- | --- | --- |
| A_CHP |  |  |  |  |
| 0.75 (0.07, 8.11) | CHOP |  |  |  |
| 1.07 (0, 357.99) | 1.41 (0.01, 287.09) | GDPT |  |  |
| 1.03 (0.03, 38.11) | 1.37 (0.09, 20.7) | 0.97 (0, 364.61) | ICED |  |
| 1 (0.03, 29.04) | 1.33 (0.12, 14.2) | 0.94 (0, 312.77) | 0.97 (0.03, 36.46) | Ro_CHOP |

Table S4 AIT Subgroup PFS League Table

| HR 95%cl | | | | |
| --- | --- | --- | --- | --- |
| A_CHP |  |  |  |  |
| 1.38 (0.06, 31.18) | CHOP |  |  |  |
| 3.13 (0.03, 292.61) | 2.26 (0.08, 60.8) | GDPT |  |  |
| 1.07 (0.01, 78.11) | 0.77 (0.04, 14.55) | 0.34 (0, 28.23) | ICED |  |
| 2.6 (0.09, 81.12) | 1.88 (0.45, 7.87) | 0.83 (0.02, 30.1) | 2.43 (0.09, 64.11) | Ro_CHOP |

Table S5 ORR League Table

| OR 95%cl | | | | | | |
| --- | --- | --- | --- | --- | --- | --- |
| A_CHP |  |  |  |  |  |  |
| 1.93 (1.23, 3.06) | CHOP |  |  |  |  |  |
| 0.98 (0.44, 2.15) | 0.51 (0.26, 0.97) | GDPT |  |  |  |  |
| 2.9 (0.95, 9.23) | 1.51 (0.54, 4.34) | 2.98 (0.89, 10.38) | GEM_P |  |  |  |
| 2.22 (0.97, 5.07) | 1.15 (0.58, 2.29) | 2.27 (0.89, 5.93) | 0.76 (0.22, 2.61) | ICED |  |  |
| 1.41 (0.83, 2.41) | 0.73 (0.55, 0.97) | 1.45 (0.71, 2.97) | 0.49 (0.16, 1.4) | 0.64 (0.3, 1.34) | Ro_CHOP |  |
| 2.8 (1.05, 7.52) | 1.45 (0.61, 3.5) | 2.87 (0.97, 8.64) | 0.96 (0.24, 3.71) | 1.26 (0.41, 3.88) | 1.98 (0.79, 5) | VIP_rABVD |

Table S6 DCR League Table

| OR 95%cl | | | | | |
| --- | --- | --- | --- | --- | --- |
| A_CHP |  |  |  |  |  |
| 1.76 (1.09, 2.88) | CHOP |  |  |  |  |
| 0.99 (0.44, 2.23) | 0.56 (0.29, 1.08) | GDPT |  |  |  |
| 2.45 (0.72, 8.64) | 1.39 (0.45, 4.46) | 2.48 (0.67, 9.42) | GEM_P |  |  |
| 2.15 (0.91, 5.09) | 1.22 (0.6, 2.49) | 2.17 (0.83, 5.73) | 0.88 (0.22, 3.35) | ICED |  |
| 1.28 (0.73, 2.28) | 0.73 (0.54, 0.98) | 1.29 (0.63, 2.67) | 0.52 (0.16, 1.69) | 0.6 (0.28, 1.28) | Ro_CHOP |

Table S7 AE Anemia League Table

| OR 95%cl | | | | |
| --- | --- | --- | --- | --- |
| A_CHP |  |  |  |  |
| 1.38 (0.85, 2.25) | CHOP |  |  |  |
| 0.89 (0.19, 3.81) | 0.65 (0.15, 2.53) | GEM_P |  |  |
| 0.15 (0.03, 0.54) | 0.11 (0.02, 0.36) | 0.16 (0.02, 1.12) | ICED |  |
| 0.33 (0.17, 0.63) | 0.24 (0.15, 0.37) | 0.37 (0.09, 1.7) | 2.19 (0.61, 10.93) | Ro_CHOP |

Table S8 Neutropenia League Table

| OR 95%cl | | | | | |
| --- | --- | --- | --- | --- | --- |
| A_CHP |  |  |  |  |  |
| 1.02 (0.06, 18.48) | CHOP |  |  |  |  |
| 2.77 (0.04, 181.22) | 2.72 (0.14, 55.89) | GEM_P |  |  |  |
| 0.38 (0.01, 24.07) | 0.37 (0.02, 7.27) | 0.14 (0, 9.23) | ICED |  |  |
| 0.51 (0.01, 31.72) | 0.5 (0.03, 9.47) | 0.19 (0, 12.68) | 1.36 (0.02, 89.03) |  |  |
| 0.08 (0, 5.66) | 0.08 (0, 1.75) | 0.03 (0, 2.07) | 0.22 (0, 15.9) | 0.16 (0, 10.99) | VIP_rABVD |

Table S9 AE Thrombocytopenia League Table

| OR 95%cl | | | | |
| --- | --- | --- | --- | --- |
| CHOP |  |  |  |  |
| 0.23 (0.06, 0.76) | GEM_P |  |  |  |
| 0.24 (0.09, 0.57) | 1.02 (0.22, 5.23) | ICED |  |  |
| 0.11 (0.06, 0.18) | 0.47 (0.13, 2.07) | 0.46 (0.17, 1.37) | Ro_CHOP |  |
| 0.02 (0.01, 0.07) | 0.1 (0.02, 0.61) | 0.1 (0.02, 0.44) | 0.21 (0.05, 0.77) | VIP_rABVD |

Supplementary material 1 Search strategy

Pubmed

Search number,Query,Sort By,Filters,Search Details,Results,Time

7,"(((""Lymphoma, T-Cell, Peripheral""[Mesh]) OR (((((((((Lymphoma, T Cell, Peripheral[Title/Abstract]) OR (Peripheral T-Cell Lymphoma[Title/Abstract])) OR (Lymphoma, Peripheral T-Cell[Title/Abstract])) OR (Lymphomas, Peripheral T-Cell[Title/Abstract])) OR (Peripheral T Cell Lymphoma[Title/Abstract])) OR (Peripheral T-Cell Lymphomas[Title/Abstract])) OR (T-Cell Lymphomas, Peripheral[Title/Abstract])) OR (T-Cell Lymphoma, Peripheral[Title/Abstract])) OR (T Cell Lymphoma, Peripheral[Title/Abstract]))) AND (((((newly diagnosed[Title/Abstract]) OR (untreated[Title/Abstract])) OR (first-line[Title/Abstract])) OR (front-line[Title/Abstract])) OR (initial[Title/Abstract]))) AND (randomized controlled trial[Publication Type] OR randomized[Title/Abstract] OR placebo[Title/Abstract])",,,"(""lymphoma, t cell, peripheral""[MeSH Terms] OR (""lymphoma t cell peripheral""[Title/Abstract] OR ""peripheral t cell lymphoma""[Title/Abstract] OR ""lymphoma peripheral t cell""[Title/Abstract] OR ""lymphomas peripheral t cell""[Title/Abstract] OR ""peripheral t cell lymphoma""[Title/Abstract] OR ""peripheral t cell lymphomas""[Title/Abstract] OR ""t cell lymphomas peripheral""[Title/Abstract] OR ""t cell lymphoma peripheral""[Title/Abstract] OR ""t cell lymphoma peripheral""[Title/Abstract])) AND (""newly diagnosed""[Title/Abstract] OR ""untreated""[Title/Abstract] OR ""first-line""[Title/Abstract] OR ""front-line""[Title/Abstract] OR ""initial""[Title/Abstract]) AND (""randomized controlled trial""[Publication Type] OR ""randomized""[Title/Abstract] OR ""placebo""[Title/Abstract])",44,02:33:43

6,randomized controlled trial[Publication Type] OR randomized[Title/Abstract] OR placebo[Title/Abstract],,,"""randomized controlled trial""[Publication Type] OR ""randomized""[Title/Abstract] OR ""placebo""[Title/Abstract]","1,114,148",02:33:22

5,"((""Lymphoma, T-Cell, Peripheral""[Mesh]) OR (((((((((Lymphoma, T Cell, Peripheral[Title/Abstract]) OR (Peripheral T-Cell Lymphoma[Title/Abstract])) OR (Lymphoma, Peripheral T-Cell[Title/Abstract])) OR (Lymphomas, Peripheral T-Cell[Title/Abstract])) OR (Peripheral T Cell Lymphoma[Title/Abstract])) OR (Peripheral T-Cell Lymphomas[Title/Abstract])) OR (T-Cell Lymphomas, Peripheral[Title/Abstract])) OR (T-Cell Lymphoma, Peripheral[Title/Abstract])) OR (T Cell Lymphoma, Peripheral[Title/Abstract]))) AND (((((newly diagnosed[Title/Abstract]) OR (untreated[Title/Abstract])) OR (first-line[Title/Abstract])) OR (front-line[Title/Abstract])) OR (initial[Title/Abstract]))",,,"(""lymphoma, t cell, peripheral""[MeSH Terms] OR (""lymphoma t cell peripheral""[Title/Abstract] OR ""peripheral t cell lymphoma""[Title/Abstract] OR ""lymphoma peripheral t cell""[Title/Abstract] OR ""lymphomas peripheral t cell""[Title/Abstract] OR ""peripheral t cell lymphoma""[Title/Abstract] OR ""peripheral t cell lymphomas""[Title/Abstract] OR ""t cell lymphomas peripheral""[Title/Abstract] OR ""t cell lymphoma peripheral""[Title/Abstract] OR ""t cell lymphoma peripheral""[Title/Abstract])) AND (""newly diagnosed""[Title/Abstract] OR ""untreated""[Title/Abstract] OR ""first-line""[Title/Abstract] OR ""front-line""[Title/Abstract] OR ""initial""[Title/Abstract])",629,02:30:59

4,((((newly diagnosed[Title/Abstract]) OR (untreated[Title/Abstract])) OR (first-line[Title/Abstract])) OR (front-line[Title/Abstract])) OR (initial[Title/Abstract]),,,"""newly diagnosed""[Title/Abstract] OR ""untreated""[Title/Abstract] OR ""first-line""[Title/Abstract] OR ""front-line""[Title/Abstract] OR ""initial""[Title/Abstract]","1,387,011",02:30:52

3,"(""Lymphoma, T-Cell, Peripheral""[Mesh]) OR (((((((((Lymphoma, T Cell, Peripheral[Title/Abstract]) OR (Peripheral T-Cell Lymphoma[Title/Abstract])) OR (Lymphoma, Peripheral T-Cell[Title/Abstract])) OR (Lymphomas, Peripheral T-Cell[Title/Abstract])) OR (Peripheral T Cell Lymphoma[Title/Abstract])) OR (Peripheral T-Cell Lymphomas[Title/Abstract])) OR (T-Cell Lymphomas, Peripheral[Title/Abstract])) OR (T-Cell Lymphoma, Peripheral[Title/Abstract])) OR (T Cell Lymphoma, Peripheral[Title/Abstract]))",,,"""lymphoma, t cell, peripheral""[MeSH Terms] OR ""lymphoma t cell peripheral""[Title/Abstract] OR ""peripheral t cell lymphoma""[Title/Abstract] OR ""lymphoma peripheral t cell""[Title/Abstract] OR ""lymphomas peripheral t cell""[Title/Abstract] OR ""peripheral t cell lymphoma""[Title/Abstract] OR ""peripheral t cell lymphomas""[Title/Abstract] OR ""t cell lymphomas peripheral""[Title/Abstract] OR ""t cell lymphoma peripheral""[Title/Abstract] OR ""t cell lymphoma peripheral""[Title/Abstract]","4,570",02:28:14

2,"((((((((Lymphoma, T Cell, Peripheral[Title/Abstract]) OR (Peripheral T-Cell Lymphoma[Title/Abstract])) OR (Lymphoma, Peripheral T-Cell[Title/Abstract])) OR (Lymphomas, Peripheral T-Cell[Title/Abstract])) OR (Peripheral T Cell Lymphoma[Title/Abstract])) OR (Peripheral T-Cell Lymphomas[Title/Abstract])) OR (T-Cell Lymphomas, Peripheral[Title/Abstract])) OR (T-Cell Lymphoma, Peripheral[Title/Abstract])) OR (T Cell Lymphoma, Peripheral[Title/Abstract])",,,"""lymphoma t cell peripheral""[Title/Abstract] OR ""peripheral t cell lymphoma""[Title/Abstract] OR ""lymphoma peripheral t cell""[Title/Abstract] OR ""lymphomas peripheral t cell""[Title/Abstract] OR ""peripheral t cell lymphoma""[Title/Abstract] OR ""peripheral t cell lymphomas""[Title/Abstract] OR ""t cell lymphomas peripheral""[Title/Abstract] OR ""t cell lymphoma peripheral""[Title/Abstract] OR ""t cell lymphoma peripheral""[Title/Abstract]","4,122",02:28:05

1,"""Lymphoma, T-Cell, Peripheral""[Mesh]",Most Recent,,"""lymphoma, t cell, peripheral""[MeSH Terms]","2,434",02:26:08

Cochrane

#1	MeSH descriptor: [Lymphoma, T-Cell, Peripheral] explode all trees	96

#2	(Lymphoma, T-Cell, Peripheral):ti,ab,kw OR (Lymphoma, T Cell, Peripheral):ti,ab,kw OR (Peripheral T-Cell Lymphoma):ti,ab,kw OR (Lymphoma, Peripheral T-Cell):ti,ab,kw OR (Lymphomas, Peripheral T-Cell):ti,ab,kw	688

#3	(Peripheral T Cell Lymphoma):ti,ab,kw OR (Peripheral T-Cell Lymphomas):ti,ab,kw OR (T-Cell Lymphomas, Peripheral):ti,ab,kw OR (T-Cell Lymphoma, Peripheral):ti,ab,kw OR (T Cell Lymphoma, Peripheral):ti,ab,kw	688

#4	#1 or #2 or #3	688

#5	(newly diagnosed):ti,ab,kw OR (untreated):ti,ab,kw OR (first-line):ti,ab,kw OR (front-line):ti,ab,kw OR (initial):ti,ab,kw	154613

#6	#4 and #5	272

Web of science

Entitlements	#	Search Query	Database	Results	Date Run

"

- WOS: 1982 to 2025

- CSCD: 1989 to 2025

- DIIDW: 1966 to 2025

- GRANTS: 1953 to 2025

- INSPEC: 1969 to 2025

- KJD: 1980 to 2025

- MEDLINE: 1950 to 2025

- PCI: 1950 to 2025

- PPRN: 1991 to 2025

- PQDT: 1637 to 2025

- SCIELO: 2002 to 2025"	1	"skeletal muscle (Topic) AND Energy metabolism (Topic) AND mitochondria (Topic) and Preprint Citation Index (Exclude – Database)	"	All Databases	8383	Tue Mar 25 2025 17:13:10 GMT+0800 (China Standard Time)

"

- WOS: 1982 to 2025

- CSCD: 1989 to 2025

- DIIDW: 1966 to 2025

- GRANTS: 1953 to 2025

- INSPEC: 1969 to 2025

- KJD: 1980 to 2025

- MEDLINE: 1950 to 2025

- PCI: 1950 to 2025

- PPRN: 1991 to 2025

- PQDT: 1637 to 2025

- SCIELO: 2002 to 2025"	2	"skeletal muscle (Topic) AND Energy metabolism (Topic) AND mitochondria (Topic) and Preprint Citation Index (Exclude – Database) and 2025 or 2024 or 2023 or 2022 or 2021 (Publication Years)	"	All Databases	1456	Tue Mar 25 2025 17:13:10 GMT+0800 (China Standard Time)

"

- WOS: 1982 to 2025

- CSCD: 1989 to 2025

- DIIDW: 1966 to 2025

- GRANTS: 1953 to 2025

- INSPEC: 1969 to 2025

- KJD: 1980 to 2025

- MEDLINE: 1950 to 2025

- PCI: 1950 to 2025

- PPRN: 1991 to 2025

- PQDT: 1637 to 2025

- SCIELO: 2002 to 2025"	3	"real world (Topic) and Preprint Citation Index (Exclude – Database)	"	All Databases	722915	Tue Mar 25 2025 17:13:12 GMT+0800 (China Standard Time)

"

- WOS: 1982 to 2025

- CSCD: 1989 to 2025

- DIIDW: 1966 to 2025

- GRANTS: 1953 to 2025

- INSPEC: 1969 to 2025

- KJD: 1980 to 2025

- MEDLINE: 1950 to 2025

- PCI: 1950 to 2025

- PPRN: 1991 to 2025

- PQDT: 1637 to 2025

- SCIELO: 2002 to 2025"	4	"real world (Topic) and Preprint Citation Index (Exclude – Database) and 2024 IEEE CVF CONFERENCE ON COMPUTER VISION AND PATTERN RECOGNITION CVPR or 2023 IEEE CVF CONFERENCE ON COMPUTER VISION AND PATTERN RECOGNITION CVPR or IEEE CVF INTERNATIONAL CONFERENCE ON COMPUTER VISION ICCV (Conferences/Meeting Titles)	"	All Databases	1208	Tue Mar 25 2025 17:13:12 GMT+0800 (China Standard Time)

"

- WOS: 1982 to 2025

- CSCD: 1989 to 2025

- DIIDW: 1966 to 2025

- GRANTS: 1953 to 2025

- INSPEC: 1969 to 2025

- KJD: 1980 to 2025

- MEDLINE: 1950 to 2025

- PCI: 1950 to 2025

- PPRN: 1991 to 2025

- PQDT: 1637 to 2025

- SCIELO: 2002 to 2025"	5	"(((((KP=(Iodine Radioisotopes)) OR KP=(Iodine)) OR AB=(Iodine-131)) OR AB=(Iodine 131)) OR AB=(Radioactive iodine)) OR AB=(radioiodine) and Preprint Citation Index (Exclude – Database)	"	All Databases	42680	Tue Mar 25 2025 17:13:13 GMT+0800 (China Standard Time)

"

- WOS: 1982 to 2025

- CSCD: 1989 to 2025

- DIIDW: 1966 to 2025

- GRANTS: 1953 to 2025

- INSPEC: 1969 to 2025

- KJD: 1980 to 2025

- MEDLINE: 1950 to 2025

- PCI: 1950 to 2025

- PPRN: 1991 to 2025

- PQDT: 1637 to 2025

- SCIELO: 2002 to 2025"	6	"spectral index (Abstract) AND som (Abstract) and Preprint Citation Index (Exclude – Database)	"	All Databases	200	Tue Mar 25 2025 17:13:27 GMT+0800 (China Standard Time)

"

- WOS: 1982 to 2025

- CSCD: 1989 to 2025

- DIIDW: 1966 to 2025

- GRANTS: 1953 to 2025

- INSPEC: 1969 to 2025

- KJD: 1980 to 2025

- MEDLINE: 1950 to 2025

- PCI: 1950 to 2025

- PPRN: 1991 to 2025

- PQDT: 1637 to 2025

- SCIELO: 2002 to 2025"	7	"RUN1 (Topic) AND rubber tree (Topic) and Preprint Citation Index (Exclude – Database)	"	All Databases	0	Tue Mar 25 2025 17:13:33 GMT+0800 (China Standard Time)

"

- WOS: 1982 to 2025

- CSCD: 1989 to 2025

- DIIDW: 1966 to 2025

- GRANTS: 1953 to 2025

- INSPEC: 1969 to 2025

- KJD: 1980 to 2025

- MEDLINE: 1950 to 2025

- PCI: 1950 to 2025

- PPRN: 1991 to 2025

- PQDT: 1637 to 2025

- SCIELO: 2002 to 2025"	8	"RUN (Topic) AND rubber tree (Topic) and Preprint Citation Index (Exclude – Database)	"	All Databases	135	Tue Mar 25 2025 17:13:39 GMT+0800 (China Standard Time)

"

- WOS: 1982 to 2025

- CSCD: 1989 to 2025

- DIIDW: 1966 to 2025

- GRANTS: 1953 to 2025

- INSPEC: 1969 to 2025

- KJD: 1980 to 2025

- MEDLINE: 1950 to 2025

- PCI: 1950 to 2025

- PPRN: 1991 to 2025

- PQDT: 1637 to 2025

- SCIELO: 2002 to 2025"	9	"giladi (Author) and Preprint Citation Index (Exclude – Database)	"	All Databases	2425	Tue Mar 25 2025 17:13:44 GMT+0800 (China Standard Time)

"

- WOS: 1982 to 2025

- CSCD: 1989 to 2025

- DIIDW: 1966 to 2025

- GRANTS: 1953 to 2025

- INSPEC: 1969 to 2025

- KJD: 1980 to 2025

- MEDLINE: 1950 to 2025

- PCI: 1950 to 2025

- PPRN: 1991 to 2025

- PQDT: 1637 to 2025

- SCIELO: 2002 to 2025"	10	"Environmental impact of DOS (Topic) and Preprint Citation Index (Exclude – Database)	"	All Databases	17131	Tue Mar 25 2025 17:13:55 GMT+0800 (China Standard Time)

"

- WOS: 1982 to 2025

- CSCD: 1989 to 2025

- DIIDW: 1966 to 2025

- GRANTS: 1953 to 2025

- INSPEC: 1969 to 2025

- KJD: 1980 to 2025

- MEDLINE: 1950 to 2025

- PCI: 1950 to 2025

- PPRN: 1991 to 2025

- PQDT: 1637 to 2025

- SCIELO: 2002 to 2025"	11	"giladi (Author) and Preprint Citation Index (Exclude – Database) and 1996 (Publication Years)	"	All Databases	23	Tue Mar 25 2025 17:13:59 GMT+0800 (China Standard Time)

"

- WOS: 1982 to 2025

- CSCD: 1989 to 2025

- DIIDW: 1966 to 2025

- GRANTS: 1953 to 2025

- INSPEC: 1969 to 2025

- KJD: 1980 to 2025

- MEDLINE: 1950 to 2025

- PCI: 1950 to 2025

- PPRN: 1991 to 2025

- PQDT: 1637 to 2025

- SCIELO: 2002 to 2025"	12	"TAM (Abstract) AND AI receptivity (Abstract) and Preprint Citation Index (Exclude – Database)	"	All Databases	0	Tue Mar 25 2025 17:14:13 GMT+0800 (China Standard Time)

"

- WOS: 1982 to 2025

- CSCD: 1989 to 2025

- DIIDW: 1966 to 2025

- GRANTS: 1953 to 2025

- INSPEC: 1969 to 2025

- KJD: 1980 to 2025

- MEDLINE: 1950 to 2025

- PCI: 1950 to 2025

- PPRN: 1991 to 2025

- PQDT: 1637 to 2025

- SCIELO: 2002 to 2025"	13	"TAM (Abstract) OR AI receptivity (Abstract) and Preprint Citation Index (Exclude – Database)	"	All Databases	38067	Tue Mar 25 2025 17:14:32 GMT+0800 (China Standard Time)

"

- WOS: 1982 to 2025

- CSCD: 1989 to 2025

- DIIDW: 1966 to 2025

- GRANTS: 1953 to 2025

- INSPEC: 1969 to 2025

- KJD: 1980 to 2025

- MEDLINE: 1950 to 2025

- PCI: 1950 to 2025

- PPRN: 1991 to 2025

- PQDT: 1637 to 2025

- SCIELO: 2002 to 2025"	14	"RUN1 (Topic) AND rubber tree (Topic) and Preprint Citation Index (Exclude – Database)	"	All Databases	0	Tue Mar 25 2025 17:14:49 GMT+0800 (China Standard Time)

"

- WOS: 1982 to 2025

- CSCD: 1989 to 2025

- DIIDW: 1966 to 2025

- GRANTS: 1953 to 2025

- INSPEC: 1969 to 2025

- KJD: 1980 to 2025

- MEDLINE: 1950 to 2025

- PCI: 1950 to 2025

- PPRN: 1991 to 2025

- PQDT: 1637 to 2025

- SCIELO: 2002 to 2025"	15	"Bioscience of Microbiota Food and Health (Publication/Source Titles) and Preprint Citation Index (Exclude – Database)	"	All Databases	315	Tue Mar 25 2025 17:15:31 GMT+0800 (China Standard Time)

"

- WOS: 1982 to 2025

- CSCD: 1989 to 2025

- DIIDW: 1966 to 2025

- GRANTS: 1953 to 2025

- INSPEC: 1969 to 2025

- KJD: 1980 to 2025

- MEDLINE: 1950 to 2025

- PCI: 1950 to 2025

- PPRN: 1991 to 2025

- PQDT: 1637 to 2025

- SCIELO: 2002 to 2025"	16	"TAM (Abstract) AND AI receptivity (Abstract) and Preprint Citation Index (Exclude – Database)	"	All Databases	0	Tue Mar 25 2025 17:15:47 GMT+0800 (China Standard Time)

"

- WOS: 1982 to 2025

- CSCD: 1989 to 2025

- DIIDW: 1966 to 2025

- GRANTS: 1953 to 2025

- INSPEC: 1969 to 2025

- KJD: 1980 to 2025

- MEDLINE: 1950 to 2025

- PCI: 1950 to 2025

- PPRN: 1991 to 2025

- PQDT: 1637 to 2025

- SCIELO: 2002 to 2025"	17	"parylene (Topic) and Preprint Citation Index (Exclude – Database)	"	All Databases	7163	Tue Mar 25 2025 17:16:54 GMT+0800 (China Standard Time)

"

- WOS: 1982 to 2025

- CSCD: 1989 to 2025

- DIIDW: 1966 to 2025

- GRANTS: 1953 to 2025

- INSPEC: 1969 to 2025

- KJD: 1980 to 2025

- MEDLINE: 1950 to 2025

- PCI: 1950 to 2025

- PPRN: 1991 to 2025

- PQDT: 1637 to 2025

- SCIELO: 2002 to 2025"	18	"(((((KP=(Iodine Radioisotopes)) OR KP=(Iodine)) OR AB=(Iodine-131)) OR AB=(Iodine 131)) OR AB=(Radioactive iodine)) OR AB=(radioiodine) and Preprint Citation Index (Exclude – Database)	Timespan: 2024-03-25 to 2025-03-25	"	All Databases	3278	Tue Mar 25 2025 17:17:17 GMT+0800 (China Standard Time)

"

- WOS: 1982 to 2025

- CSCD: 1989 to 2025

- DIIDW: 1966 to 2025

- GRANTS: 1953 to 2025

- INSPEC: 1969 to 2025

- KJD: 1980 to 2025

- MEDLINE: 1950 to 2025

- PCI: 1950 to 2025

- PPRN: 1991 to 2025

- PQDT: 1637 to 2025

- SCIELO: 2002 to 2025"	19	"TAM (Abstract) OR Artificial Intelligence (Abstract) AND Intelligence (Abstract) and Preprint Citation Index (Exclude – Database)	"	All Databases	454727	Tue Mar 25 2025 17:17:30 GMT+0800 (China Standard Time)

"

- WOS: 1982 to 2025

- CSCD: 1989 to 2025

- DIIDW: 1966 to 2025

- GRANTS: 1953 to 2025

- INSPEC: 1969 to 2025

- KJD: 1980 to 2025

- MEDLINE: 1950 to 2025

- PCI: 1950 to 2025

- PPRN: 1991 to 2025

- PQDT: 1637 to 2025

- SCIELO: 2002 to 2025"	20	"TS=(Lymphoma, T-Cell, Peripheral) OR TS=(Lymphoma, T Cell, Peripheral) and Preprint Citation Index (Exclude – Database)	"	All Databases	24858	Tue Mar 25 2025 17:17:43 GMT+0800 (China Standard Time)

"

- WOS: 1982 to 2025

- CSCD: 1989 to 2025

- DIIDW: 1966 to 2025

- GRANTS: 1953 to 2025

- INSPEC: 1969 to 2025

- KJD: 1980 to 2025

- MEDLINE: 1950 to 2025

- PCI: 1950 to 2025

- PPRN: 1991 to 2025

- PQDT: 1637 to 2025

- SCIELO: 2002 to 2025"	21	"TS=(Lymphoma, T-Cell, Peripheral) OR TS=(Lymphoma, T Cell, Peripheral) and Preprint Citation Index (Exclude – Database)	"	All Databases	24858	Tue Mar 25 2025 17:18:06 GMT+0800 (China Standard Time)

"

- WOS: 1982 to 2025

- CSCD: 1989 to 2025

- DIIDW: 1966 to 2025

- GRANTS: 1953 to 2025

- INSPEC: 1969 to 2025

- KJD: 1980 to 2025

- MEDLINE: 1950 to 2025

- PCI: 1950 to 2025

- PPRN: 1991 to 2025

- PQDT: 1637 to 2025

- SCIELO: 2002 to 2025"	22	"TAM (Abstract) OR Artificial Intelligence (Abstract) AND Intelligence (Abstract) and Preprint Citation Index (Exclude – Database) and Social Sciences or Technology (Research Domains)	"	All Databases	389890	Tue Mar 25 2025 17:18:19 GMT+0800 (China Standard Time)

"

- WOS: 1982 to 2025

- CSCD: 1989 to 2025

- DIIDW: 1966 to 2025

- GRANTS: 1953 to 2025

- INSPEC: 1969 to 2025

- KJD: 1980 to 2025

- MEDLINE: 1950 to 2025

- PCI: 1950 to 2025

- PPRN: 1991 to 2025

- PQDT: 1637 to 2025

- SCIELO: 2002 to 2025"	23	"Ascertaining the validity of individual protocols from web-based personality inventories. (Topic) and Preprint Citation Index (Exclude – Database)	"	All Databases	1	Tue Mar 25 2025 17:18:37 GMT+0800 (China Standard Time)

"

- WOS: 1982 to 2025

- CSCD: 1989 to 2025

- DIIDW: 1966 to 2025

- GRANTS: 1953 to 2025

- INSPEC: 1969 to 2025

- KJD: 1980 to 2025

- MEDLINE: 1950 to 2025

- PCI: 1950 to 2025

- PPRN: 1991 to 2025

- PQDT: 1637 to 2025

- SCIELO: 2002 to 2025"	24	"TS=(Lymphoma, T-Cell, Peripheral) OR TS=(Lymphoma, T Cell, Peripheral) OR TS=(Peripheral T-Cell Lymphoma) OR TS=(Lymphoma, Peripheral T-Cell) and Preprint Citation Index (Exclude – Database)	"	All Databases	24858	Tue Mar 25 2025 17:19:10 GMT+0800 (China Standard Time)

"

- WOS: 1982 to 2025

- CSCD: 1989 to 2025

- DIIDW: 1966 to 2025

- GRANTS: 1953 to 2025

- INSPEC: 1969 to 2025

- KJD: 1980 to 2025

- MEDLINE: 1950 to 2025

- PCI: 1950 to 2025

- PPRN: 1991 to 2025

- PQDT: 1637 to 2025

- SCIELO: 2002 to 2025"	25	"TS=(Lymphoma, T-Cell, Peripheral) OR TS=(Lymphoma, T Cell, Peripheral) OR TS=(Peripheral T-Cell Lymphoma) OR TS=(Lymphoma, Peripheral T-Cell) OR TS=(Lymphomas, Peripheral T-Cell ) OR TS=(Peripheral T Cell Lymphoma) OR TS=(Peripheral T-Cell Lymphomas) OR TS=(T-Cell Lymphomas, Peripheral) OR TS=(T-Cell Lymphoma, Peripheral) OR TS=(T Cell Lymphoma, Peripheral) and Preprint Citation Index (Exclude – Database)	"	All Databases	24858	Tue Mar 25 2025 17:19:33 GMT+0800 (China Standard Time)

"

- WOS: 1982 to 2025

- CSCD: 1989 to 2025

- DIIDW: 1966 to 2025

- GRANTS: 1953 to 2025

- INSPEC: 1969 to 2025

- KJD: 1980 to 2025

- MEDLINE: 1950 to 2025

- PCI: 1950 to 2025

- PPRN: 1991 to 2025

- PQDT: 1637 to 2025

- SCIELO: 2002 to 2025"	26	"(((((AB=(Myelosuppression)) OR AB=(marrow suppression)) OR AB=(bone marrow suppression)) OR AB=(complete blood count)) OR AB=(complete blood count changes)) OR AB=(abnormal blood) and Preprint Citation Index (Exclude – Database)	Timespan: 2024-03-25 to 2025-03-25	"	All Databases	10465	Tue Mar 25 2025 17:19:34 GMT+0800 (China Standard Time)

"

- WOS: 1982 to 2025

- CSCD: 1989 to 2025

- DIIDW: 1966 to 2025

- GRANTS: 1953 to 2025

- INSPEC: 1969 to 2025

- KJD: 1980 to 2025

- MEDLINE: 1950 to 2025

- PCI: 1950 to 2025

- PPRN: 1991 to 2025

- PQDT: 1637 to 2025

- SCIELO: 2002 to 2025"	27	"Atmospheric evolution of emissions from a boreal forest fire: the formation of highly functionalized oxygen-, nitrogen-, and sulfur-containing organic compounds (Topic) and Preprint Citation Index (Exclude – Database)	"	All Databases	2	Tue Mar 25 2025 17:19:53 GMT+0800 (China Standard Time)

"

- WOS: 1982 to 2025

- CSCD: 1989 to 2025

- DIIDW: 1966 to 2025

- GRANTS: 1953 to 2025

- INSPEC: 1969 to 2025

- KJD: 1980 to 2025

- MEDLINE: 1950 to 2025

- PCI: 1950 to 2025

- PPRN: 1991 to 2025

- PQDT: 1637 to 2025

- SCIELO: 2002 to 2025"	28	"#5 AND #26 and Preprint Citation Index (Exclude – Database)	Timespan: 2024-03-25 to 2025-03-25	"	All Databases	10	Tue Mar 25 2025 17:20:19 GMT+0800 (China Standard Time)

"

- WOS: 1982 to 2025

- CSCD: 1989 to 2025

- DIIDW: 1966 to 2025

- GRANTS: 1953 to 2025

- INSPEC: 1969 to 2025

- KJD: 1980 to 2025

- MEDLINE: 1950 to 2025

- PCI: 1950 to 2025

- PPRN: 1991 to 2025

- PQDT: 1637 to 2025

- SCIELO: 2002 to 2025"	29	"TAM (Abstract) AND Intelligence (Abstract) and Preprint Citation Index (Exclude – Database) and Social Sciences or Technology (Research Domains)	"	All Databases	399	Tue Mar 25 2025 17:21:18 GMT+0800 (China Standard Time)

"

- WOS: 1982 to 2025

- CSCD: 1989 to 2025

- DIIDW: 1966 to 2025

- GRANTS: 1953 to 2025

- INSPEC: 1969 to 2025

- KJD: 1980 to 2025

- MEDLINE: 1950 to 2025

- PCI: 1950 to 2025

- PPRN: 1991 to 2025

- PQDT: 1637 to 2025

- SCIELO: 2002 to 2025"	30	"spectral index (Abstract) AND som (Abstract) AND sentinel (Abstract) and Preprint Citation Index (Exclude – Database)	"	All Databases	20	Tue Mar 25 2025 17:21:19 GMT+0800 (China Standard Time)

"

- WOS: 1982 to 2025

- CSCD: 1989 to 2025

- DIIDW: 1966 to 2025

- GRANTS: 1953 to 2025

- INSPEC: 1969 to 2025

- KJD: 1980 to 2025

- MEDLINE: 1950 to 2025

- PCI: 1950 to 2025

- PPRN: 1991 to 2025

- PQDT: 1637 to 2025

- SCIELO: 2002 to 2025"	31	"Identifying careless responses in survey data. (Topic) and Preprint Citation Index (Exclude – Database)	"	All Databases	62	Tue Mar 25 2025 17:21:21 GMT+0800 (China Standard Time)

"

- WOS: 1982 to 2025

- CSCD: 1989 to 2025

- DIIDW: 1966 to 2025

- GRANTS: 1953 to 2025

- INSPEC: 1969 to 2025

- KJD: 1980 to 2025

- MEDLINE: 1950 to 2025

- PCI: 1950 to 2025

- PPRN: 1991 to 2025

- PQDT: 1637 to 2025

- SCIELO: 2002 to 2025"	32	"TS=(newly diagnosed) OR TS=(untreated) OR TS=(first-line) OR TS=(front-line) OR TS=(initial) and Preprint Citation Index (Exclude – Database)	"	All Databases	4307560	Tue Mar 25 2025 17:22:06 GMT+0800 (China Standard Time)

"

- WOS: 1982 to 2025

- CSCD: 1989 to 2025

- DIIDW: 1966 to 2025

- GRANTS: 1953 to 2025

- INSPEC: 1969 to 2025

- KJD: 1980 to 2025

- MEDLINE: 1950 to 2025

- PCI: 1950 to 2025

- PPRN: 1991 to 2025

- PQDT: 1637 to 2025

- SCIELO: 2002 to 2025"	33	"TAM (Abstract) AND Literacy (Abstract) and Preprint Citation Index (Exclude – Database) and Social Sciences or Technology (Research Domains)	"	All Databases	175	Tue Mar 25 2025 17:22:12 GMT+0800 (China Standard Time)

"

- WOS: 1982 to 2025

- CSCD: 1989 to 2025

- DIIDW: 1966 to 2025

- GRANTS: 1953 to 2025

- INSPEC: 1969 to 2025

- KJD: 1980 to 2025

- MEDLINE: 1950 to 2025

- PCI: 1950 to 2025

- PPRN: 1991 to 2025

- PQDT: 1637 to 2025

- SCIELO: 2002 to 2025"	34	"Smith (Author) AND frost (Topic) and Preprint Citation Index (Exclude – Database)	"	All Databases	264	Tue Mar 25 2025 17:22:49 GMT+0800 (China Standard Time)

"

- WOS: 1982 to 2025

- CSCD: 1989 to 2025

- DIIDW: 1966 to 2025

- GRANTS: 1953 to 2025

- INSPEC: 1969 to 2025

- KJD: 1980 to 2025

- MEDLINE: 1950 to 2025

- PCI: 1950 to 2025

- PPRN: 1991 to 2025

- PQDT: 1637 to 2025

- SCIELO: 2002 to 2025"	35	"TAM (Abstract) AND Literacy (Abstract) and Preprint Citation Index (Exclude – Database)	"	All Databases	197	Tue Mar 25 2025 17:25:03 GMT+0800 (China Standard Time)

"

- WOS: 1982 to 2025

- CSCD: 1989 to 2025

- DIIDW: 1966 to 2025

- GRANTS: 1953 to 2025

- INSPEC: 1969 to 2025

- KJD: 1980 to 2025

- MEDLINE: 1950 to 2025

- PCI: 1950 to 2025

- PPRN: 1991 to 2025

- PQDT: 1637 to 2025

- SCIELO: 2002 to 2025"	36	"TAM (Abstract) AND Literacy (Abstract)	"	All Databases	197	Tue Mar 25 2025 17:25:03 GMT+0800 (China Standard Time)

"

- WOS: 1982 to 2025

- CSCD: 1989 to 2025

- DIIDW: 1966 to 2025

- GRANTS: 1953 to 2025

- INSPEC: 1969 to 2025

- KJD: 1980 to 2025

- MEDLINE: 1950 to 2025

- PCI: 1950 to 2025

- PPRN: 1991 to 2025

- PQDT: 1637 to 2025

- SCIELO: 2002 to 2025"	37	"TAM (Abstract) AND Literacy (Abstract)	"	All Databases	197	Tue Mar 25 2025 17:25:14 GMT+0800 (China Standard Time)

"

- WOS: 1982 to 2025

- CSCD: 1989 to 2025

- DIIDW: 1966 to 2025

- GRANTS: 1953 to 2025

- INSPEC: 1969 to 2025

- KJD: 1980 to 2025

- MEDLINE: 1950 to 2025

- PCI: 1950 to 2025

- PPRN: 1991 to 2025

- PQDT: 1637 to 2025

- SCIELO: 2002 to 2025"	38	"TS=(randomized controlled trial) OR TS=(randomized) OR TS=(placebo) OR TS=(random) OR TS=(double-blind) and Preprint Citation Index (Exclude – Database)	"	All Databases	3954744	Tue Mar 25 2025 17:25:48 GMT+0800 (China Standard Time)

"

- WOS: 1982 to 2025

- CSCD: 1989 to 2025

- DIIDW: 1966 to 2025

- GRANTS: 1953 to 2025

- INSPEC: 1969 to 2025

- KJD: 1980 to 2025

- MEDLINE: 1950 to 2025

- PCI: 1950 to 2025

- PPRN: 1991 to 2025

- PQDT: 1637 to 2025

- SCIELO: 2002 to 2025"	39	"#38 AND #32 AND #25 and Preprint Citation Index (Exclude – Database)	"	All Databases	258	Tue Mar 25 2025 17:26:15 GMT+0800 (China Standard Time)

Embase

No.,Query,Results,Date

#22,"#11 AND #17 AND #21",43,25 Mar 2025

#21,"#18 OR #19 OR #20",943859,25 Mar 2025

#20,"'double-blind':ab,ti",238383,25 Mar 2025

#19,"'placebo':ab,ti",391637,25 Mar 2025

#18,"'random':ab,ti",490251,25 Mar 2025

#17,"#12 OR #13 OR #14 OR #15 OR #16",2008361,25 Mar 2025

#16,"'initial':ab,ti",1420495,25 Mar 2025

#15,"'front-line':ab,ti",15502,25 Mar 2025

#14,"'first-line':ab,ti",217588,25 Mar 2025

#13,"'untreated':ab,ti",294684,25 Mar 2025

#12,"'newly diagnosed':ab,ti",133330,25 Mar 2025

#11,"#1 OR #2 OR #3 OR #4 OR #5 OR #6 OR #7 OR #8 OR #9 OR #10",35684,25 Mar 2025

#10,"'t cell lymphoma, peripheral':ab,ti",24,25 Mar 2025

#9,"'t-cell lymphoma, peripheral':ab,ti",24,25 Mar 2025

#8,"'t-cell lymphomas, peripheral':ab,ti",8,25 Mar 2025

#7,"'peripheral t-cell lymphomas':ab,ti",2112,25 Mar 2025

#6,"'peripheral t cell lymphoma':ab,ti",5499,25 Mar 2025

#5,"'lymphomas, peripheral t-cell':ab,ti",27,25 Mar 2025

#4,"'lymphoma, peripheral t-cell':ab,ti",99,25 Mar 2025

#3,"'peripheral t-cell lymphoma':ab,ti",5499,25 Mar 2025

#2,"'lymphoma, t cell, peripheral':ab,ti",1,25 Mar 2025

#1,"'peripheral t cell lymphoma'/exp",34386,25 Mar 2025
